# Circular Melt-Spun Textile Fibers from Polyethylene-like Long-Chain Polyesters

**DOI:** 10.1021/acsapm.4c01570

**Published:** 2024-07-30

**Authors:** Katrin Wurst, Melissa Birkle, Katharina J. Scherer, Stefan Mecking

**Affiliations:** Department of Chemistry, University of Konstanz, Universitätsstrasse 10, 78457 Konstanz, Germany

**Keywords:** biodegradability, closed-loop recycling, long-chain polyester, melt-spinning, synthetic fiber

## Abstract

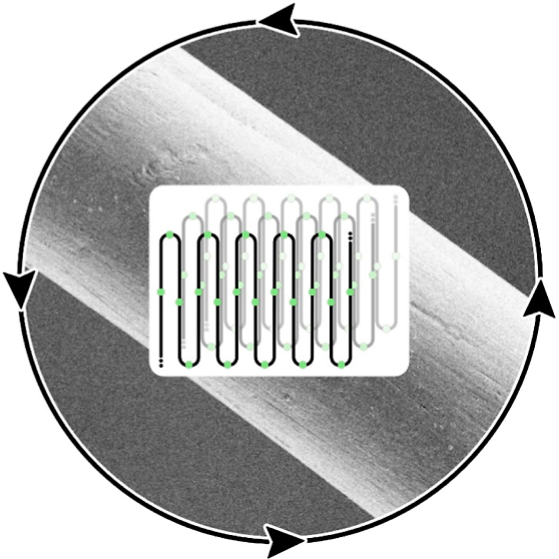

As textiles contribute considerably to overall anthropogenic pollution and resource consumption, increasing their circularity is essential. We report the melt-spinning of long-chain polyesters, materials recently shown to be fully chemically recyclable under mild conditions, as well as biodegradable. High-quality uniform fibers are enabled by the polymers’ favorable combination of thermal stability, crystallization ability, melt strength, and homogeneity. The polyethylene-like crystalline structure endows these fibers with mechanical strength, which is further enhanced by its orientation upon drawing (tensile strength of up to 270 MPa). In vitro depolymerization by high concentrations of *Humicola insolens* cutinase underlines the accessibility of the fibers for enzymatic degradation, which can proceed from the surface and through the entire fiber within days, depending on the choice of the fiber material. Fibers and knitted fabrics withstand stress, as encountered in machine washing.

## Introduction

Fibers of both natural and synthetic origins are employed on a very large scale in textiles and a multitude of further applications. Over the entire life cycle of typical fiber applications, the overall profile of resource consumptions and emissions is generally more favorable for synthetic fibers.^1,2^ Yet, synthetic fibers also face major challenges concerning their sustainability, and resolving these issues is key to achieving a circular carbon and plastics economy.^3−7^ Poly(ethylene terephthalate) (PET) is the largest fiber by scale and, thus, a relevant reference point.^8^ Its petrochemistry-derived aromatic repeat units result in a high melting point (*T*_m_ = 268 °C), which may be beneficial for some specialized applications but also renders processing and recycling more energy-consuming. PET if lost to the environment is biodegraded very slowly at best. This may be related to its above-ambient glass transition temperature, which hinders accessibility of the amorphous regimes for enzymatic breakdown.^9−11^ This is particularly relevant for textile applications because fibers abraded during washing of textiles are a major contributor to microplastics pollution.^12,13^ Other types of synthetic fibers employed for textiles, like polyamides, polyethylene (HDPE), or polypropylene (PP), also offer no benefits over PET in terms of circularity and environmental persistence. Beyond these polymers, poly(lactide) (PLA) as a biobased polyester has also been established as a fiber material, and further, biodegradable polyesters that are most prominently applied in films and packaging plastics have been studied in the form of fibers [like poly(butylene-adipate-*co*-terephthalate), PBAT; poly(hydroxy-butyrate-*co*-valerate), PHBV; and polycaprolactone, PCL].^14,15^ However, their overall profile of processability and fiber performance, in many instances, remains unsatisfactory. Often, their rather soft or brittle nature is problematic already.

Achieving a sustainable circular fibers economy requires materials beyond the established petrochemistry based plastics that (1) are designed for recycling from the outset, (2) do not persist for decades or centuries in the environment, (3) encompass strong intermolecular crystalline interactions that provide fiber strength, and (4) are compatible with melt-spinning and further processing. Long-chain aliphatic polyesters with polyethylene-like solid-state structures have been found to provide the favorable materials and processing properties of polyethylene (HDPE) in injection molding or 3D printing. At the same time, they are amenable to closed-loop recycling by solvolysis under comparatively mild conditions of 120–150 °C and can be fully biodegradable, as found recently.^16,17^

We now reveal long-chain polyesters are fully compatible with melt-spinning as the most established and environmentally friendly production process for synthetic fibers and report properties of resulting fibers and textiles produced thereof.

## Experimental Section

### Materials

All chemicals were used as received without further purification. 1,18-Octadecanedioic acid was acquired from *Elevance Renewable Sciences*, and hexamethylenediamine (≥99.5%) was acquired from *Carl Roth GmbH*. Enzyme HiC was purchased as a Novozym 51032 solution from *ChiralVision*. Tetrachloroethane-*d*_2_ was acquired from *deutero GmbH*. Phenol was purchased from *Merck*. Deionized water was employed to prepare buffer solutions for the enzyme experiments. Polyester-18,18 and polyester-2,18 were prepared analogous to reported procedures^16,17^ from 1,18-octadecanedioic acid and 1,18-octadecane diol and ethylene glycol, respectively. All polymerizations involving air- and moisture-sensitive compounds were carried out under an inert gas atmosphere using standard Schlenk techniques.

### Synthesis of Polyamide

A 285 mL Limbo reactor by *BüchiGlasUster* equipped with a glass inlet lined with polytetrafluoroethylene (PTFE) foil by *BOLA* was loaded with 1,18-octadecanedioic acid and hexamethylenediamine (1.03 equiv. vs acid). The reactor was evacuated and purged with nitrogen three times, pressurized with nitrogen to 10 bar, and heated to 160 °C. Over the course of 2.25 h, the temperature was increased to 220 °C, and this temperature was held for another 1.25 h. The reactor was vented to ambient pressure, and vacuum (6 × 10^–2^ mbar) was applied for 5 h by means of a rotary vane oil pump, while the temperature was increased to 230 °C. The reactor was cooled to room temperature, and the polymer was recovered. For melt-spinning, the material was vitrified with liquid nitrogen and broken into pieces, which were cryo-milled in an ultra centrifugal mill ZM 200 by *Retsch GmbH* with a mesh size of 1 mm. To remove condensed water, the powder was dried under vacuum at 50 °C.

### Polymer Characterization and Processing Techniques

Size exclusion chromatography (SEC) analysis of PE-2,18 was performed in chloroform employing a *PSS SECcurity*^*2*^ instrument equipped with *PSS SDV Linear M* columns and a refractive index detector *PSS SECcurity*^*2*^*RI*. Narrowly distributed polystyrene standards were employed for calibration. Molecular weight analysis of PE-18,18 employed high-temperature SEC as previously reported.^16^ Data were evaluated with *PSS WinGPC* software.

High-pressure liquid chromatography (HPLC) measurements were conducted with a *Rezex RHM-monosaccharide* H^+^ 300 × 7.80 mm 8 μm ion exchange column (*Phenomenex*). Sulfuric acid (30 mM) with a flow rate of 0.6 mL min^–1^ was used as a mobile phase at 40 °C. Detection of compounds occurred on a refractive index detector RID-10A.

Light microscopy images were recorded on a *Leica DM 4000 M* instrument equipped with a 10× objective in bright field and transmission modes. Distance measurements on light microscopy images were performed with the *Leica Application Suite* (version 4.13.0) software.

Nuclear magnetic resonance (NMR) spectra were recorded on a *Bruker Avance III HD 400* spectrometer. ^1^H chemical shifts were referenced to the solvent signals (C_2_D_2_Cl_4_ = 6 ppm). Multiplicities are reported as s (singlet), d (doublet), t (triplet), q (quartet), quint (quintet), and m (multiplet). Data evaluation was conducted on the *Mestrelab Research S.L*. (version 14.3.1) software.

Scanning electron microscopy (SEM) images were recorded on a *Zeiss Gemini 500* instrument with a SE2 and an InLens detector at an acceleration voltage of 1 kV on nonsputtered samples.

Tensile testing of fibers was performed according to ISO 11566 on a *ZwickRoell 1446 RetroLine tC II* instrument with a 5 N load cell. Specimens 2 cm in length were fastened into the sample holders coated with an elastomeric material by tightening the spring screws. Measurements commenced at an elongation rate of 1 mm min^–1^ for determination of the modulus and proceeded at an elongation rate of 50 mm min^–1^ until rupture of the specimen. Fibers were analyzed by testing 3–7 specimens, and standard deviations were calculated accordingly.

Wide-angle X-ray scattering (WAXS) analysis was performed on a *Bruker AXS D8 Discover* diffractometer equipped with a IμS microfocus X-ray source (Cu–Kα radiation) and a two-dimensional *VÅNTEC-500 Area* detector.

The linear density of fibers was determined from weight and length measurements of groups of a bundle of fibers. The linear density was calculated from the total length and weight of the fibers.

### Melt-Spinning and Fiber Processing Procedures

Melt-spinning of monofilaments was conducted on a *Xplore MC15 HT* twin screw micro compounder supplied by a feeder and equipped with a 1.5 mm die (3 mm for polyamide) and a winding unit of the *Xplore Fiber Line* placed at a distance of 1 m from each other. Polymer granulate was dispensed at a defined dosing speed into the micro compounder, operated at 120 °C for PE-2,18 and PE-18,18 and at 180 °C for HDPE. Polyamide-6,18 (PA-6,18) was melt-spun by the batchwise addition of polymer powder followed by melting and extruding at 210 °C.

Drawing of the PE-2,18 monofilament was conducted on the *Conditioning Unit* of the *Xplore Fiber Line*. Transportation of the fiber from the supply roll by the slower rotating godet to the faster rotating godet stretched the fiber to a length determined by the difference in the speeds of the godets. Movement through a heating device warmed the fiber to 40 °C for 20–35 s during the stretching.

PE-2,18 multifilament was produced at the Deutsche Institute für Textil- and Faserforschung (DITF, Denkendorf). Polymer granulate was extruded and melt-spun by a *Reimotec GmbH* extruder connected to a customized spinneret. The fiber was coated with a commercial avivage prior to collection on a bobbin by a godet using a *Barmag SW-Wickler*. A *Zinser 548* was used to stretch the multifilament with a temperature of the godets of 60 °C and the drawing zone of 70 °C. Fabric was produced by circular knitting of PE-2,18 multifilament on a *Harry Lucas GmbH & Co. KG* knitting machine with a gauge of E24 and a diameter of 3.5 ″. Cotton fiber (79 wt %, 714 dtex) and PE-2,18 multifilament (21 wt %, 194 dtex) were twisted to produce a blended yarn on an *Agteks DirecTwist D6″-C6″* with a processing speed of 14 m min^–1^ at 400 twists per meter in the *S*-direction.

### Enzymatic Hydrolysis of Fibers

Enzymatic hydrolysis of PE-2,18 fibers was conducted as previously reported for powders^17^ employing PE-2,18 monofilament pieces with a length of approximately 1 cm. The formation of ethylene glycol was quantified by samples taken after 0, 1, 2, 4, and 8 h and 1, 2, 4, 8, and 15 d by syringe filtration. Samples for SEM analysis of the fiber surface were taken at 0, 1, 2, 4, and 8 h and 1, 2, 4, and 8 days and washed with deionized water.

### Home Laundering Experiment

Samples of PE-2,18 monofilament of 1 m length were sewn in nylon bags with a *Pfaff edition 130* sewing machine and conventional PET yarn. The edges of approximately 1 × 2 cm rectangles of PE-2,18 fabric were hemmed and placed in a nylon bag, which was sewn up. PE-18,18 monofilament samples were prepared accordingly as a reference. The samples were placed in two conventional laundry bags made from PET and tied up with a string made from a yarn mixture. The washing was conducted with a *Siemens iQ 390* washing machine using the default program for delicate laundry. 20 mL of commercial laundry detergent *Fein & Woll Waschbalsam* from *Frosch (Mainzer Werner & Mertz GmbH)* was added to each washing cycle at 30 °C, and 20 mL of *Voll-Waschmittel Citrus* from *Frosch (Mainzer Werner & Mertz GmbH)* was added to each washing cycle at 60 °C. Washing commenced at 600 rpm for 40 min at 30 °C and at 1400 rpm for 2.5 h at 60 °C for each cycle of the respective series. The samples were dried in air overnight.

## Results and Discussion

### Single Filaments

**Figure 1:**
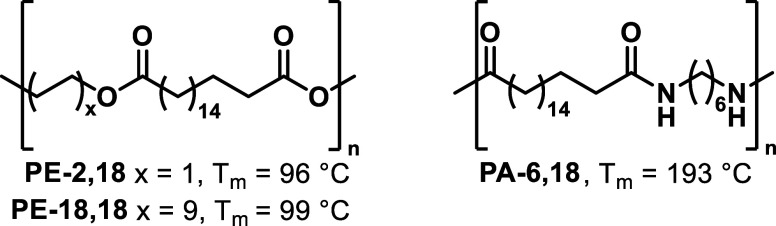
Structures of polymers studied and peak melting points.

We chose polyester-18,18^16^ (PE-18,18, Figure 1) for preliminary melt-spinning studies, among others, due to its known high crystallization rate.^18^ Spinning of PE-18,18, obtained from polycondensation of 1,18-octadecane dicarboxylic acid and 1,18-octadecanediol [*M*_w_ ≈ 188.000 g mol^–1^ and *M*_n_ ≈ 102.000 g mol^–1^ as determined by SEC vs polystyrene standards, cf. Figure S2] from a melt compounder into passive ambient air without further measures afforded uniform flexible fibers (cf. the Experimental Section for details of the spinning instrumentation and conditions and analytical techniques).

**Figure 2:**
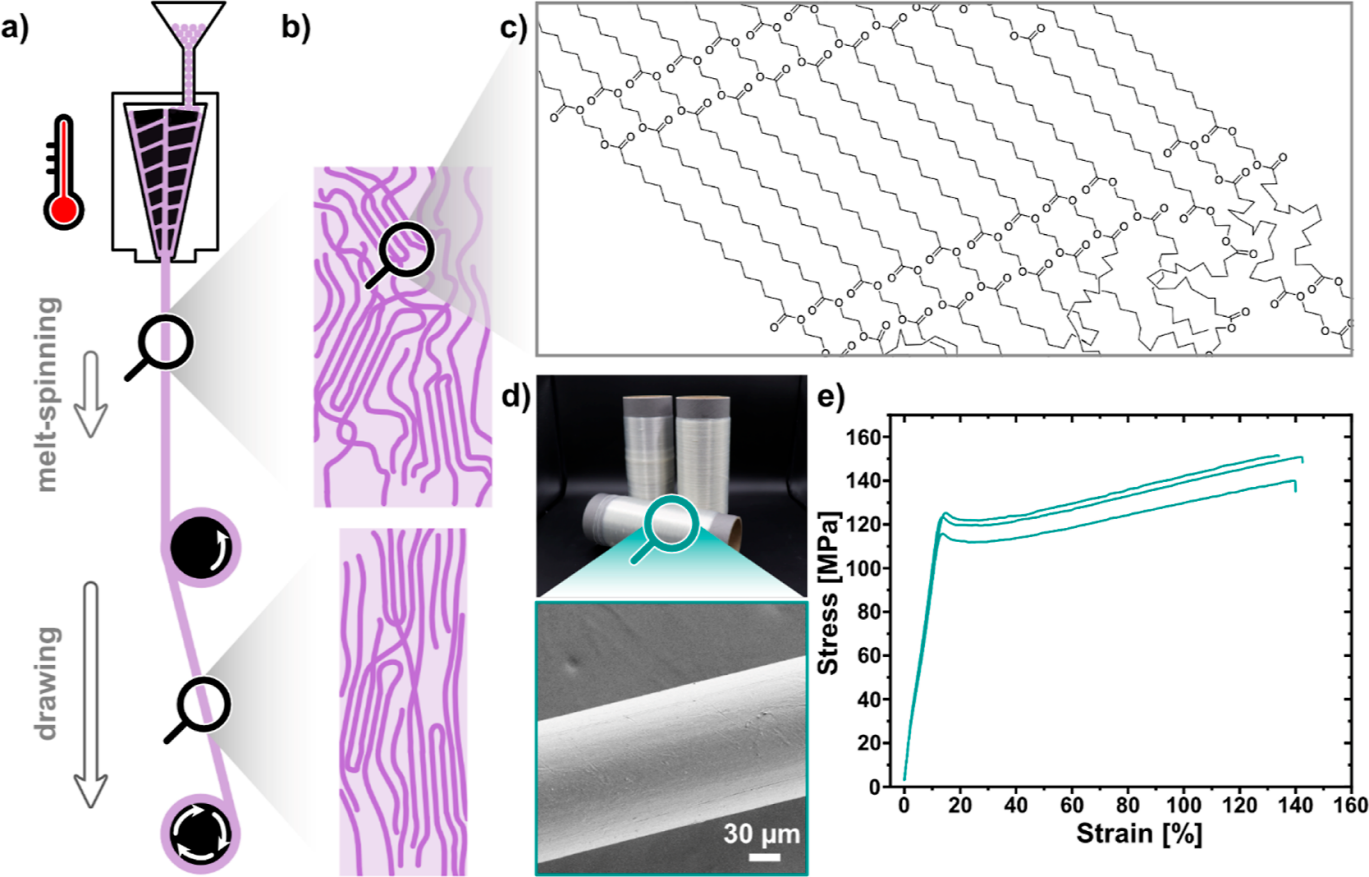
(a) Schematic representation of the melt-spinning and fiber drawing process. (b) Schematic representation of crystalline and amorphous regions in the filament of as-spun and stretched fibers. (c) Arrangement of PE-2,18 chains in crystallites and the amorphous region. (d) Spooled PE-2,18 fibers on the bobbin (top) and SEM image of a fiber (bottom). (e) Stress–strain curves of PE-2,18 fibers stretched with a draw ratio of 1:3.

No adverse effects like sharkskin formation, development of stick–slip discontinuity (so-called “bambooing”), or occurrence of melt fractures (“corkscrewing”), problems frequently observed in melt-spinning,^14,19,20^ were observed. The smooth surface and the fact that this was achieved even without extensive optimization of conditions underline the suitability of PE-18,18 for melt-spinning (cf. Figure S1). A strength of σ_b_ ≈ 53 ± 2 MPa was determined by tensile tests on the fibers (cf. Figure S3). Encouraged by these findings, polyester-2,18 (PE-2,18) was studied in depth, this material being accessible from entirely commercially available monomers and displaying an enhanced biodegradability in compost and soil compared to PE-18,18.^17^ Homogeneity of the polymer melt for optimal fiber spinning conditions was ensured by feeding with granulated PE-2,18 (*M*_w_ ≈ 160.000 g mol^–1^ and *M*_n_ ≈ 70.000 g mol^–1^, cf. Figure S10). Melt-spinning requires a melt strength that is sufficiently high to prevent filament breakage during processing but also a melt that is not too viscous to impair processability.^14^ A melt temperature of 120 °C was found to provide an optimum balance for the fiber formation process here, at winding speeds up to 160 m min^–1^ (the studied upper limit of the laboratory scale setup used, cf. Figure 2a and the Supporting Information). SEC of the fibers shows no significant alteration compared to the virgin polyester (cf. Figure S14 and Table S2). This underlines the thermal stability of the material during the melt-spinning process. Neither undesirable molecular weight breakdown nor cross-linking occurs.

The melt-spun PE-2,18 fibers possess a smooth and uniform surface as showcased by SEM micrographs (Figure 2d). Uniform fiber diameters were also observed by quantitative analysis of light microscopy images, in the range of 255 ± 2 to 57 ± 4 μm, depending on the winding speed during melt-spinning (10–160 m min^–1^. These dimensions correspond to a linear density of, e.g., 55 dtex for a fiber spun at a winding speed of 100 m min^–1^). The tensile strength of the fibers increases with the winding speed, from 54 ± 7 to 112 ± 16 MPa, along with a reduced elongation at break. This indicates the orientation already in the melt-spinning process (Figure 2b), as also confirmed by X-ray diffraction patterns obtained on a two-dimensional detector (Figure S12 in the Supporting Information). Increase of the tensile strength of the fibers due to an enhanced orientation of the polymer chains along the fiber axis was implemented by cold-drawing of the as-spun fibers (cf. the Supporting Information for details). Drawing of fibers to three times their original length doubled their tensile strength to 148 ± 7 MPa (from 74 ± 4 for the as-spun fiber), with the elongation at break reduced and the modulus of elasticity not significantly affected (Figure 2e, cf. the Supporting Information for details, Table S3, and Figure S15. A linear density of 73 dtex was determined for the drawn fiber). The molecular weight is not significantly altered by the drawing process either, as evidenced by SEC analysis (cf. Figure S16 and Table S4).

**Figure 3:**
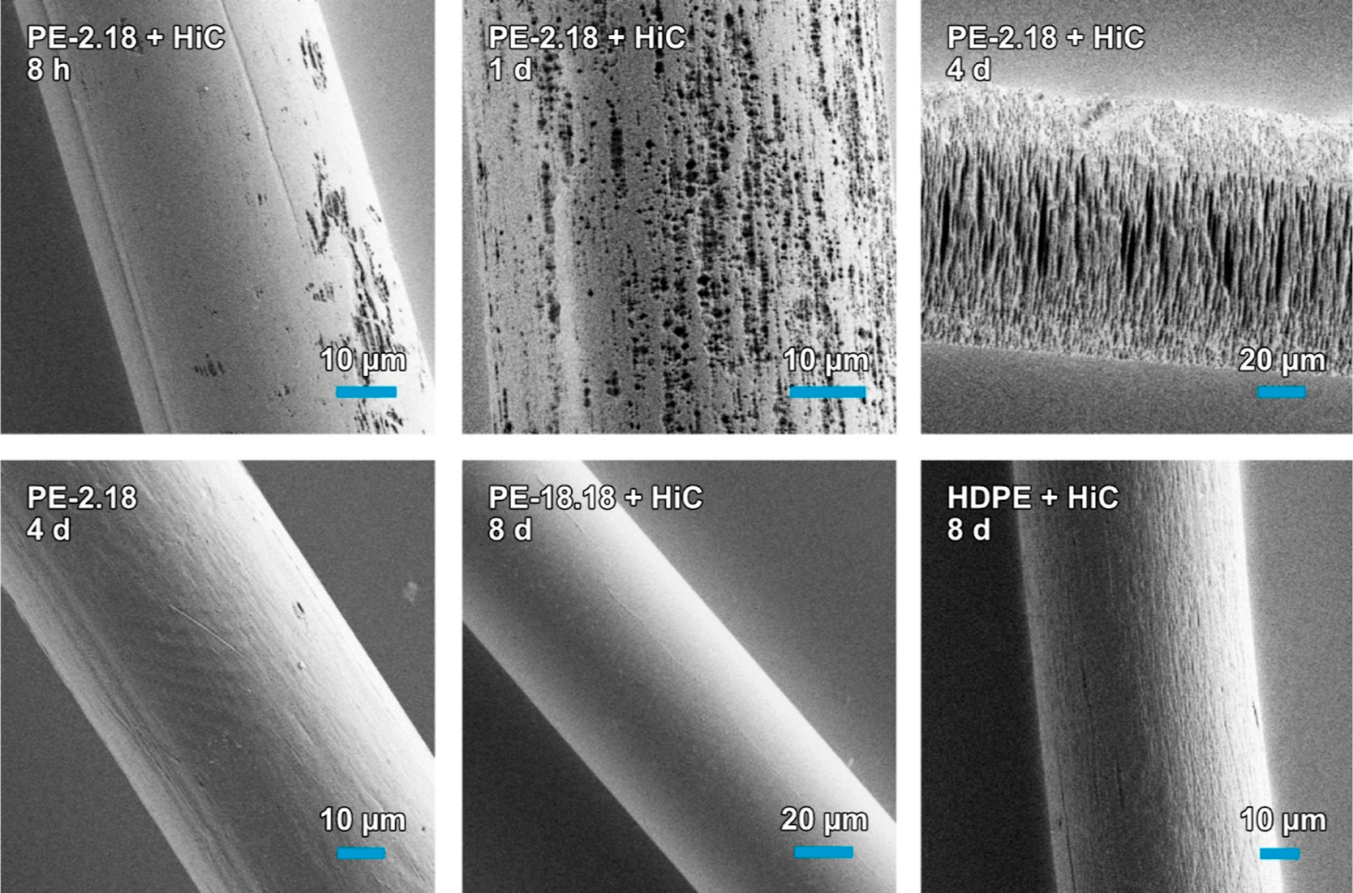
SEM images of PE-2,18 fibers after exposure to high concentrations of *Humicola insolens* cutinase (HiC). Increasingly severe surface erosion is evident after 8 h (top left), 1 day (top center), and 4 days (top right). Reference of PE-2,18 fiber subjected to the protocol but without the addition of HiC after 4 days (bottom left) and PE-18,18 fiber exposed to HiC after 8 days (bottom center). SEM image of the reference HDPE fiber after exposure to HiC for 8 days (bottom right).

The action of enzymes on drawn PE-2,18 fibers was monitored by SEM employing a protocol for exposure to the naturally occurring esterase *Humicola insolens* cutinase (HiC) previously established^17^ for polymer powders. This comprises in vitro exposure to high concentrations of HiC at 37 °C, which provides an accelerated degradation (cf. the Supporting Information for details). The absolute rates observed are not quantitatively representative of biodegradation in real-life environments such as, for example, a sewage plant or soil. Rather, these experiments are an indication of the accessibility of the fibers for enzymatic degradation and of the relative rates for different fiber materials. Already after 8 h of exposure, erosion of the fiber surface by HiC had clearly occurred (Figure 3, top left), and cavities and holes appeared over the entire fiber surface within 1 day (Figure 3, top center). These eventually propagate through the entire fiber (Figure 3, top right). After 8 days, the fibers were mostly fragmented into smaller pieces, while a reference sample treated identically but in the absence of the enzyme is unaffected (Figure 3, bottom left). Treatment of the PE-2,18 fibers with HiC solution for 15 d fragmented all discernible fibers. By strong contrast, the PE-18,18 fiber is unaffected by the HiC exposure, like the HDPE reference (Figure 3, bottom center and right, Figure S18).

### Multifilaments and Fabrics

The processability of polyester-2,18 was further probed by spinning to multifilament fibers under conditions more closely resembling those of industrial fiber production. A multifilament was spun from the melt (170 °C) with 1500 m min^–1^ to provide 20 strands with a diameter of 30 ± 1 μm of the individual strands. Note that a commercial coating (avivage) was applied to the fibers’ surface to reduce static charging and friction, enable higher velocities, and reduce abrasion during further processing. Stretching of the as-spun filament with a draw ratio of 1:2 at 60–70 °C reduced the strand’s diameter to 22 ± 1 μm. As for the PE-2,18 fibers spun as single filaments, X-ray diffraction reveals partial orientation (cf. Figure S19). Tensile testing on single strands removed from the multifilament for this purpose showed an increase of the strength from 162 ± 35 to 267 ± 66 MPa (cf. Figure S21, Table S5) upon drawing (with a linear density of 194 and 102 dtex, respectively, for the multifilament). The enhanced strength of the multifilament compared to that of the fibers spun as single filaments on a laboratory scale can be related to an increased orientation of the polymer chains in the direction of the fiber axis by spinning at much higher winding speeds (1500 vs 100 m min^–1^). The fibers’ strength compares favorably to that of PET fibers spun at a similar rate (140–220 MPa).^19,21^

**Figure 4:**
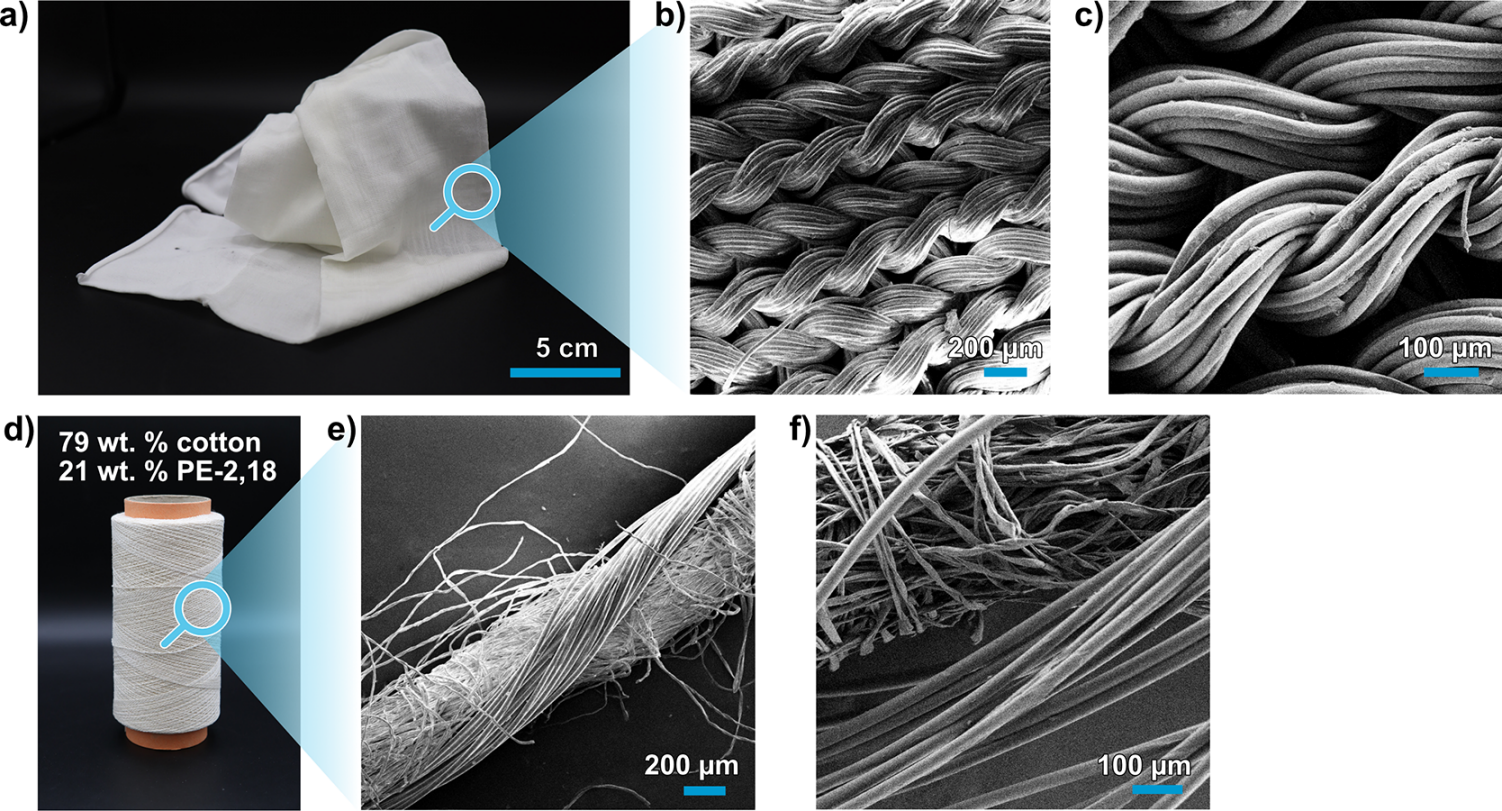
(a) Knitted fabric from the PE-2,18 multifilament. (b,c) SEM images of the PE-2,18 fabric in different magnifications. (d) Blended yarn from the mixture of 79 wt % cotton fiber and 21 wt % PE-2,18 multifilament. (e,f) SEM images of the blended yarn in different magnifications.

Furthermore, twisting of cotton fibers with the polyester multifilament yielded a blended yarn of 79 wt % cotton and 21 wt % PE-2,18 (Figure 4d) as commonly used for textile production (cf. the Experimental Section for details).^22^ Processing to textiles is enabled by the fibers’ flexibility and uniformity,^23^ as further exemplified by circular knitting of the PE-2,18 multifilament. The obtained white woven fabric displayed a sheen and a pleasant feel to the touch (Figure 4a, cf. Supporting Information Figure S22 for the knit structure. Fabric weight: 73 ± 14 g m^–2^, thickness: 313 ± 14 μm).

**Figure 5:**
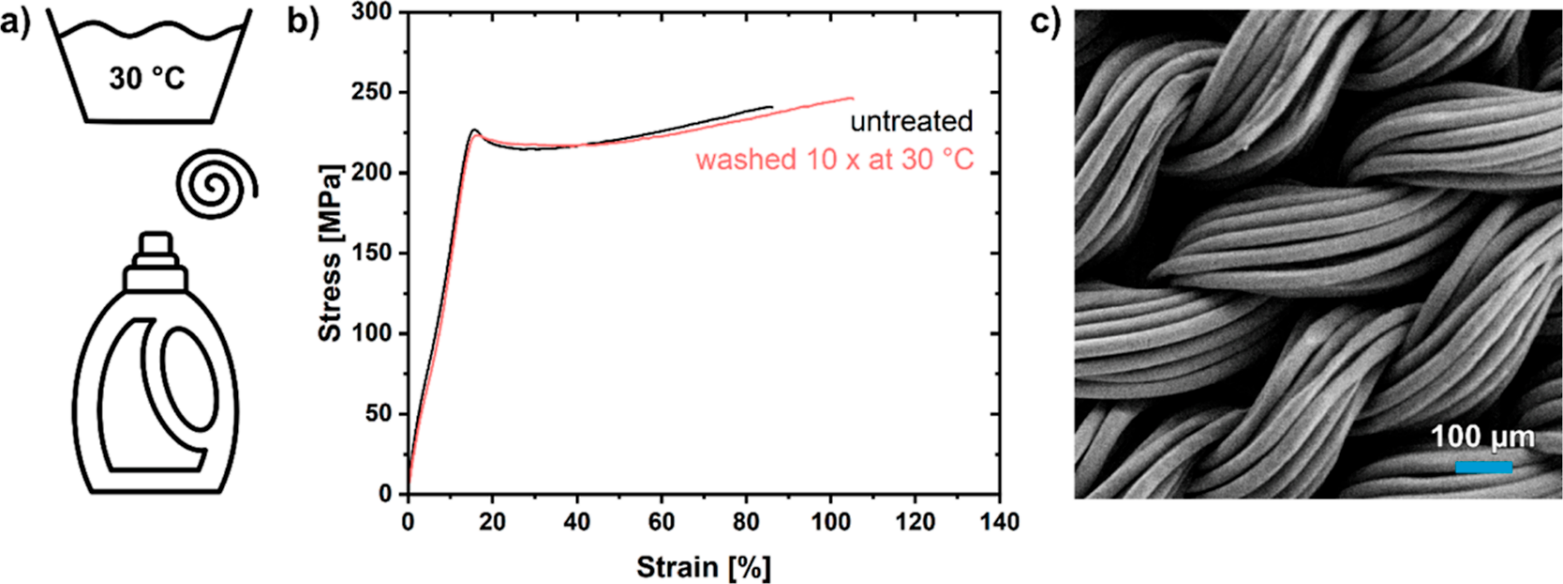
(a) Laundry program applied to PE-2,18 fibers and fabric for 10 cycles. (b) Stress–strain curves of washed and untreated PE-2,18 fibers. (c) SEM image of the PE-2,18 fabric after 10 cycles of washing.

The ability of the materials to withstand the mechanical stress of common machine washing and their compatibility with laundry detergents were probed with PE-2,18 fibers as well as fabrics (Figure 5a). Exposure of fibers to 10 full 30 °C washing cycles resulted in no observable alteration of their tensile properties (Figure 5b, see the Supporting Information for details). By comparison, after 10 full washing cycles at 60 °C, a slight decrease of the modulus was found. At both washing temperatures, the individual fibers and the fabrics remained fully intact and no damage to the fiber surface or the fabric was evident from electron microscopy, optical microscopy, and visual inspection; also, SEC analyses show that polymer molecular weights are not affected (Figure 5c and Supporting Information, Figures S26 and S27).

Preliminary studies show that the utility of long-chain polycondensates^24,25^ for melt-spun fibers extends beyond these polyesters. Polyamide-6,18, generated from polycondensation of hexamethylene diamine with 1,18-octanedicarboxylic acid with *M*_n_ ≈ 15.000 g mol^–1^ (from NMR end group quantification) and *T*_m_ = 193 °C, *T*_c_ = 174 °C could be melt-spun at 210 °C to continuous and uniform fibers (cf. the Supporting Information for details of synthesis and characterization data). Tensile testing of a fiber of 68 μm diameter showed a tensile strength of σ_b_ = 129 ± 20 MPa and an elongation at break of ε_b_ = 160 ± 40% (cf. Figure S32).

## Conclusions

Our findings demonstrate the potential of long-chain aliphatic polyesters as much sought-after circular fiber materials. The observed excellent compatibility with melt-spinning processes can be traced to the fulfillment of several general key requirements:^14^ (1) A consistent melt flow rheology is ensured, which can be related to the reasonably low polydispersity index (<3) achieved by the well-behaved nature of the polycondensation procedure employed for the polymers’ synthesis (*M*_w_/*M*_n_ ≈ 2.0–2.3). (2) The melt strength is sufficiently high to prevent filament breakage during processing, but the melt is not too viscous to impair processability, a balance achieved by an appropriate molecular weight of the material. (3) A sufficient thermal stability of the polymers to withstand the extrusion temperature and shear strain during processing without significant degradation or cross-linking, as also evidenced by molecular weights being unaffected by processing. (4) A uniform nature of the polymers and the absence of impurities that would clog the processing equipment and cause fluctuations in the processing conditions, as also evidenced by the fibers’ smooth surface and uniform diameters. (5) A facile orientation and crystallization facilitated by the strictly linear chains’ ability to unfold and align along the strain direction, as also evidenced by increasing strength and orientation upon drawing.

Further, the crystallization temperatures and rates are sufficiently high to allow for straightforward efficient melt-spinning with conventional equipment, comprising passive air cooling. Most notably, the polyethylene-like crystalline structure provides satisfactory fiber strength, which can be further significantly enhanced by orientation. The fibers generated at the maximum spinning speed studied compare favorably in their tensile strength to PET fibers spun at a similar speed.^19,21^ Despite their semicrystalline nature and rather hydrophobic, largely hydrocarbon, composition, the fibers are amenable to enzymatic degradation. The strong variability of enzymatic degradation rates with the choice of polyester repeat units, namely, the diol monomer length, offers the possibility of balancing the desirable hydrolytic stability during wear and care and a slow but sufficient biodegradation rate of fibers lost to the environment.

Notably, Carother’s seminal paper of the first synthetic fibers and the phenomenon of cold-drawing employed a long-chain aliphatic polyester, polyester-3,16, drawn from the melt with a glass rod.^26^ While this work can be considered as the foundation of today’s fiber technology, the material itself received no further interest, likely due to lack of availability of the monomers. Today’s emergence of long-chain polyesters as biobased, recyclable, and biodegradable polyethylene-like thermoplastics is enabled by the advent of commercial sources of long-chain dicarboxylates, and future prospects comprise their production from third-generation feedstocks such as microalgae or postconsumer polyethylene waste.^27,28^ The latter can be converted to (ultra) long-chain dicarboxylic acid mixtures by oxidation^29−31^ or by dehydrogenation/follow-up reactions.^28,32−34^

Our findings warrant further studies of other long-chain polyesters as textile materials, comprehensive exploration of yarns’ production, application testing of textiles, and their subsequent chemical recycling, among others.

## Supporting Information Available

Additional characterization of PE-2,18, PE-18,18, and PA-6,18 single fibers, PE-2,18 multifilament, and fabric (SEC, tensile testing, X-ray diffraction, electron microscopy, and optical microscopy) and additional data for enzymatic hydrolysis and machine washing experiments (electron microscopy, tensile testing, HPLC, and SEC) (PDF)
