# Supplementary material for: Circular Melt-Spun Textile Fibers from Polyethylene-like Long-Chain Polyesters
Source: ACS Appl Polym Mater. 2024 Jul 30;6(15):9219–25. doi: 10.1021/acsapm.4c01570 (PMC11320378; doi:10.1021/acsapm.4c01570)
Supplement: Supplementary file 1 — ap4c01570_si_001.pdf [file ap4c01570_si_001.pdf]

**Supporting Information**

# Circular Melt-Spun Textile Fibers from Polyethylene-Like Long-Chain Polyesters

*Katrin Wurst, Melissa Birkle, Katharina J. Scherer, and Stefan Mecking**

Department of Chemistry, Chair of Chemical Materials Science, University of Konstanz,
Universitätsstrasse 10, 78457 Konstanz, Germany
* stefan.mecking@uni-konstanz.de, +49(0)7531 88-5151

**Table of Contents**

S1. Additional characterization data for single filaments ........ 3
Supplementary data for PE-18,18 fibers ........ 3
Supplementary data for PE-2,18 fibers ........ 12
Supplementary data for enzymatic hydrolysis experiment ........ 19
S2. Additional characterization data for PE-2,18 multifilament ........ 21
S3. Additional characterization data for PE-2,18 fabric ........ 24
S4. Supplementary data for machine washing experiments ........ 26
S5. Additional characterization data for PA-6,18 ........ 31

## S1. Additional characterization data for single filaments

**Supplementary data for PE-18,18 fibers**

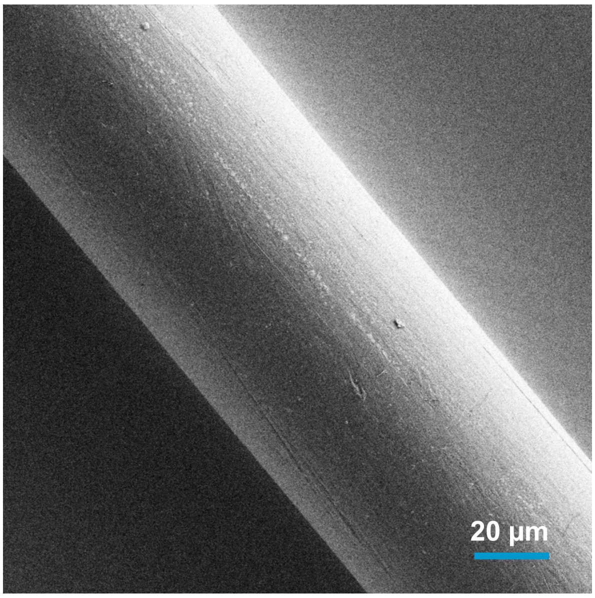


**Figure S1.** SEM image of a PE-18,18 fiber.

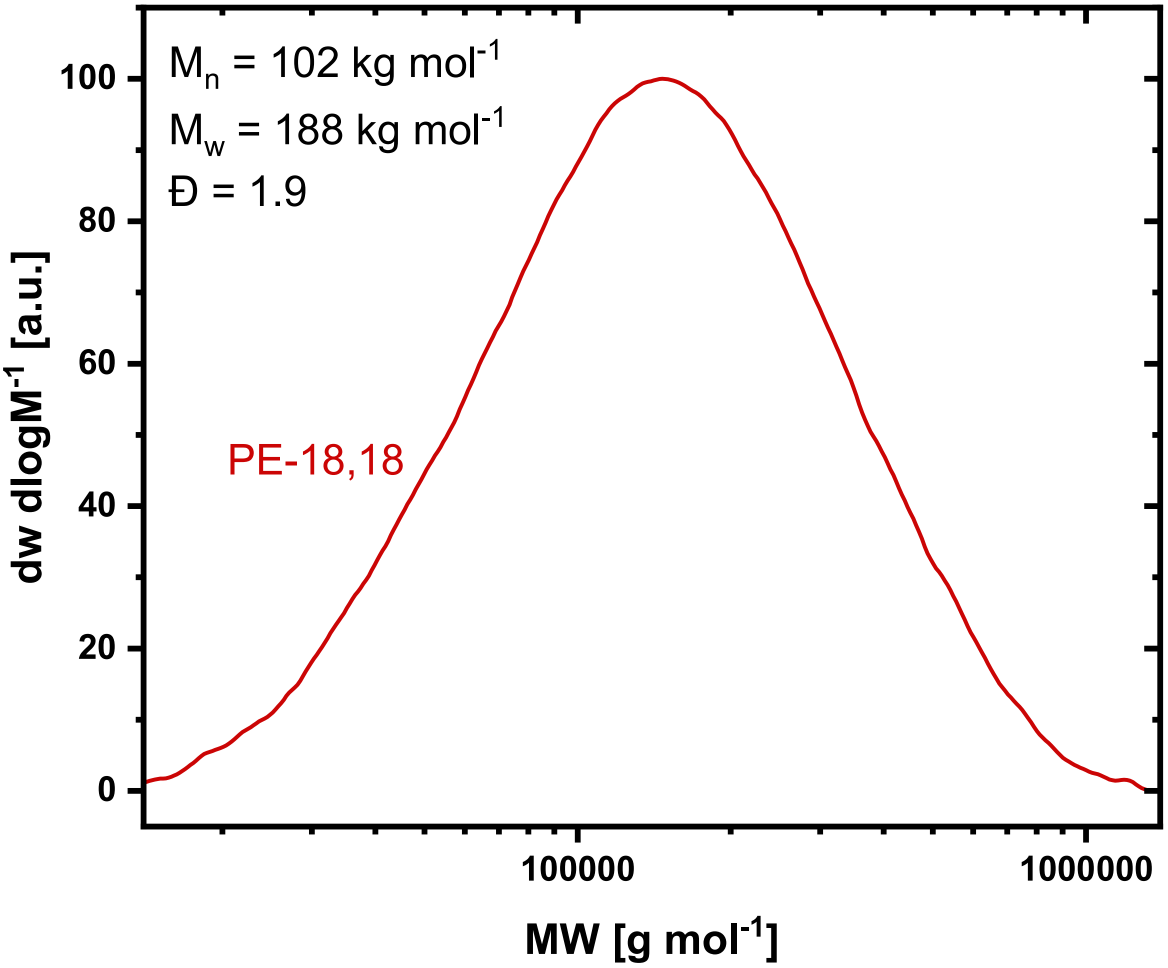


**Figure S2.** SEC trace of PE-18,18 polymer used for melt-spinning of fibers employing a *Xplore MC15 HT* micro compounder and a winding unit of the *Xplore Fiber Line*.

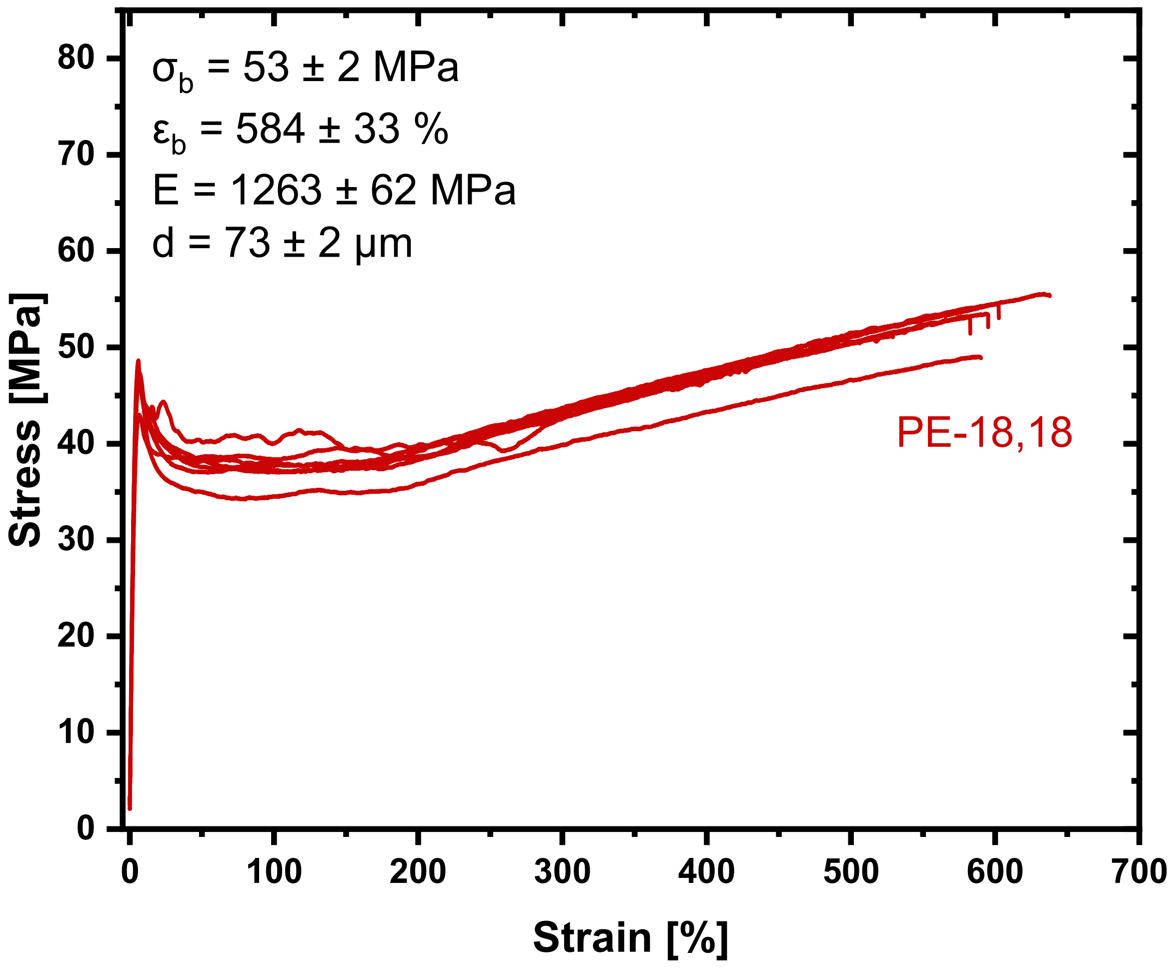


**Figure S3.** Stress-strain curves of PE-18,18 fibers.

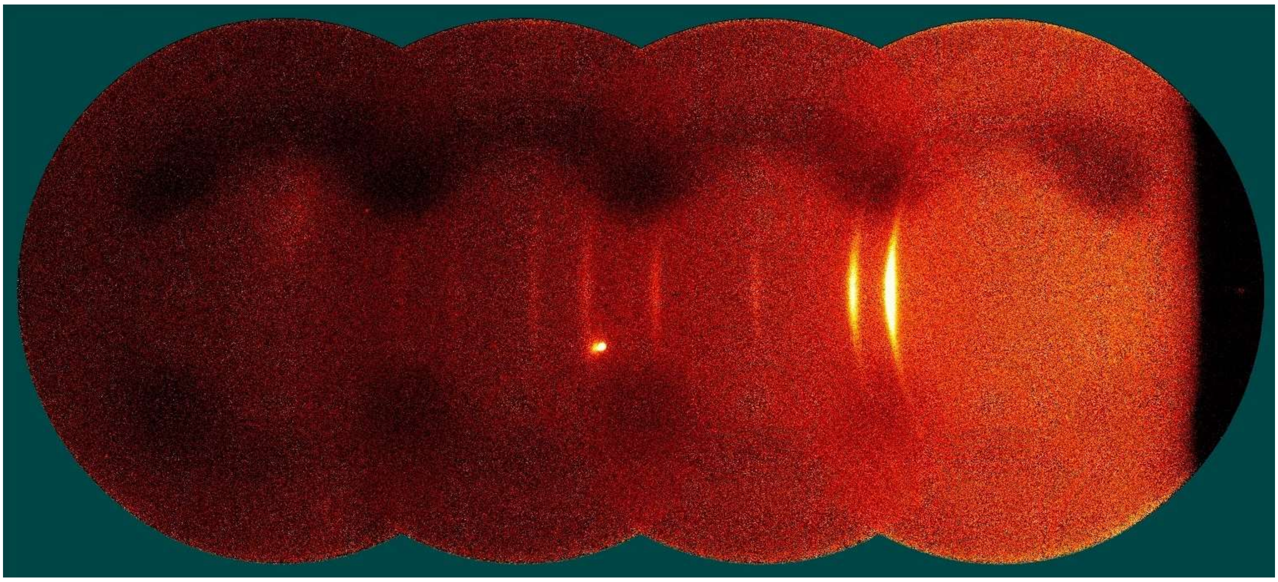

**Figure S4.** Diffraction pattern of a PE-18,18 fiber from 2D detected WAXS.

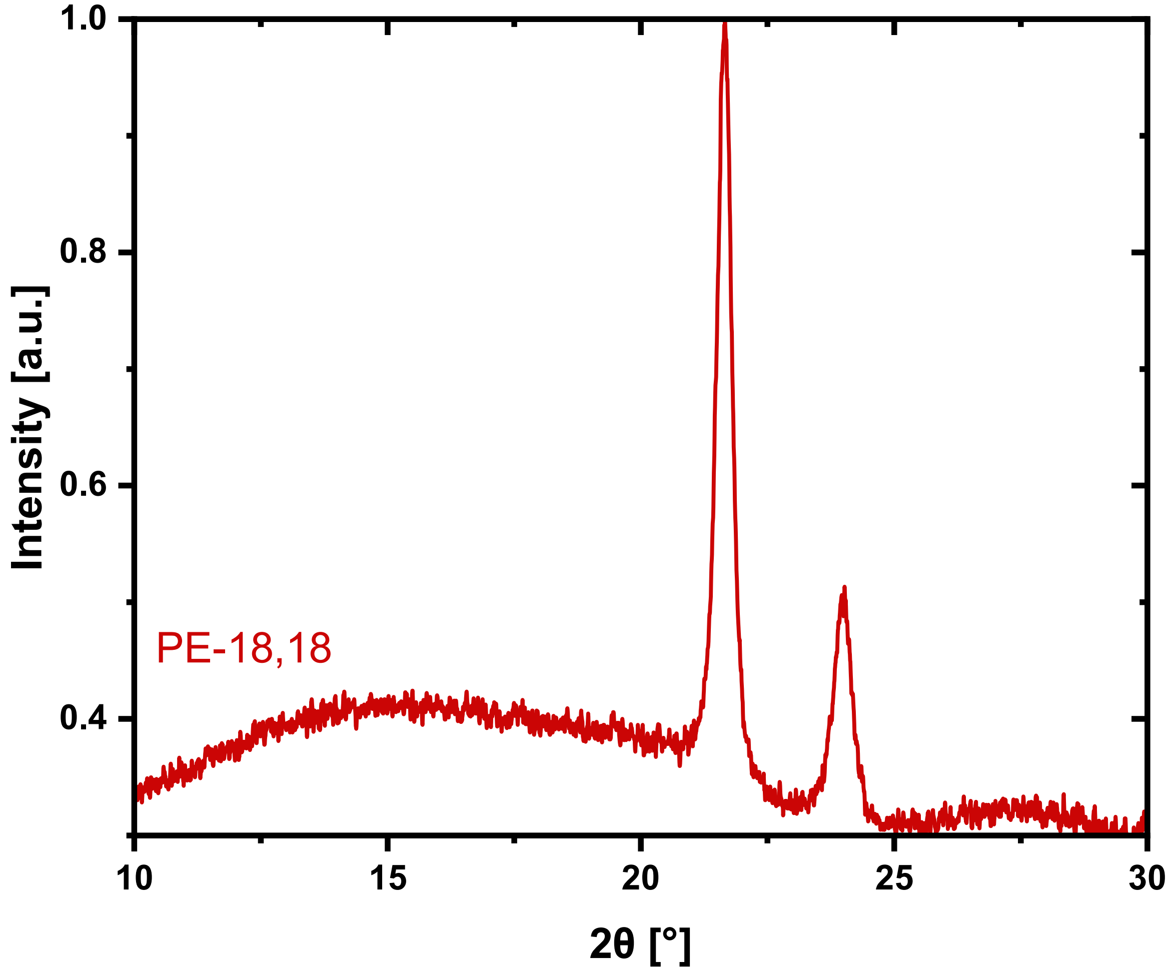


**Figure S5.** WAXS diffraction pattern of a PE-18,18 fiber.

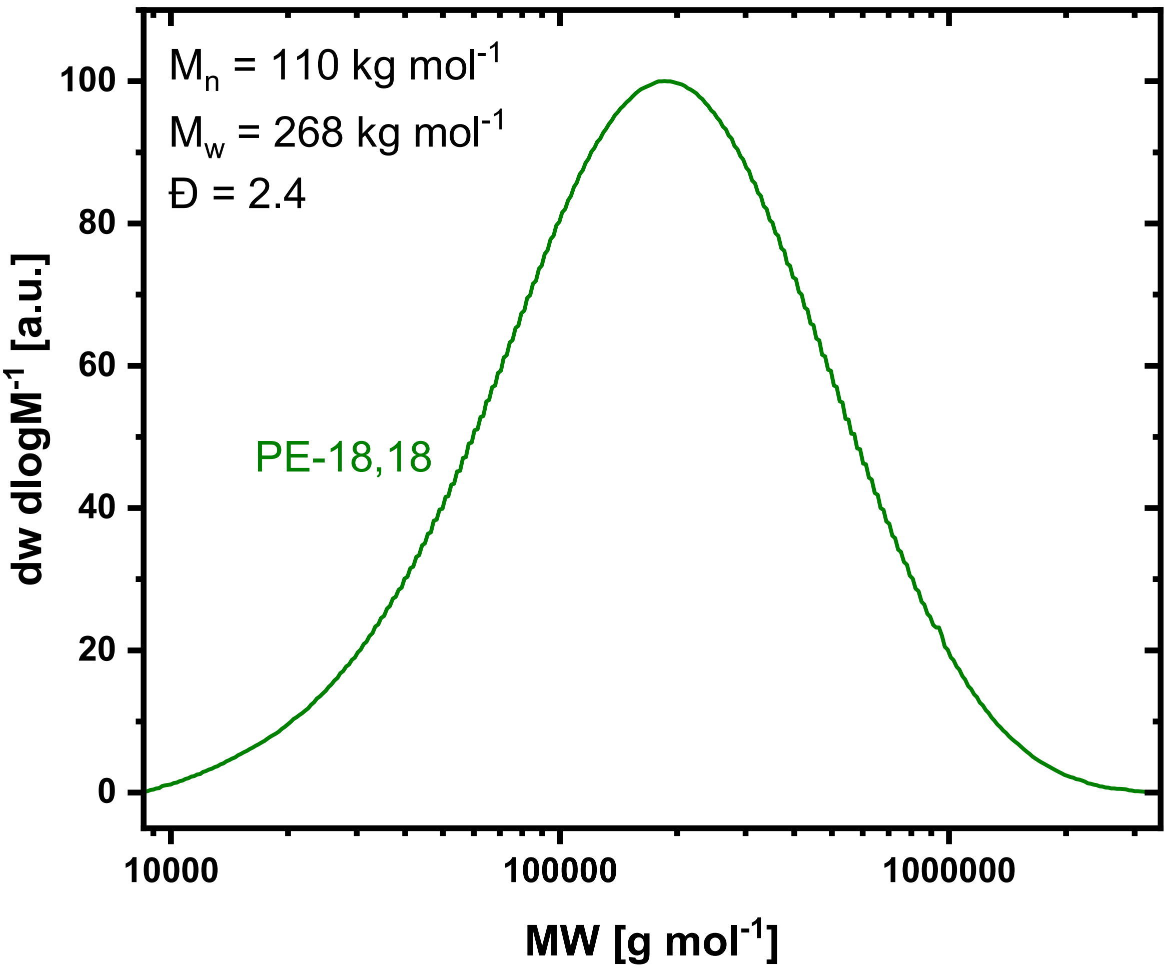


**Figure S6.** SEC trace (160 °C; 1,2-dichlorobenzene) of PE-18,18 employed for melt-spinning of fibers with a *Xplore CFL 35* micro cast film line.

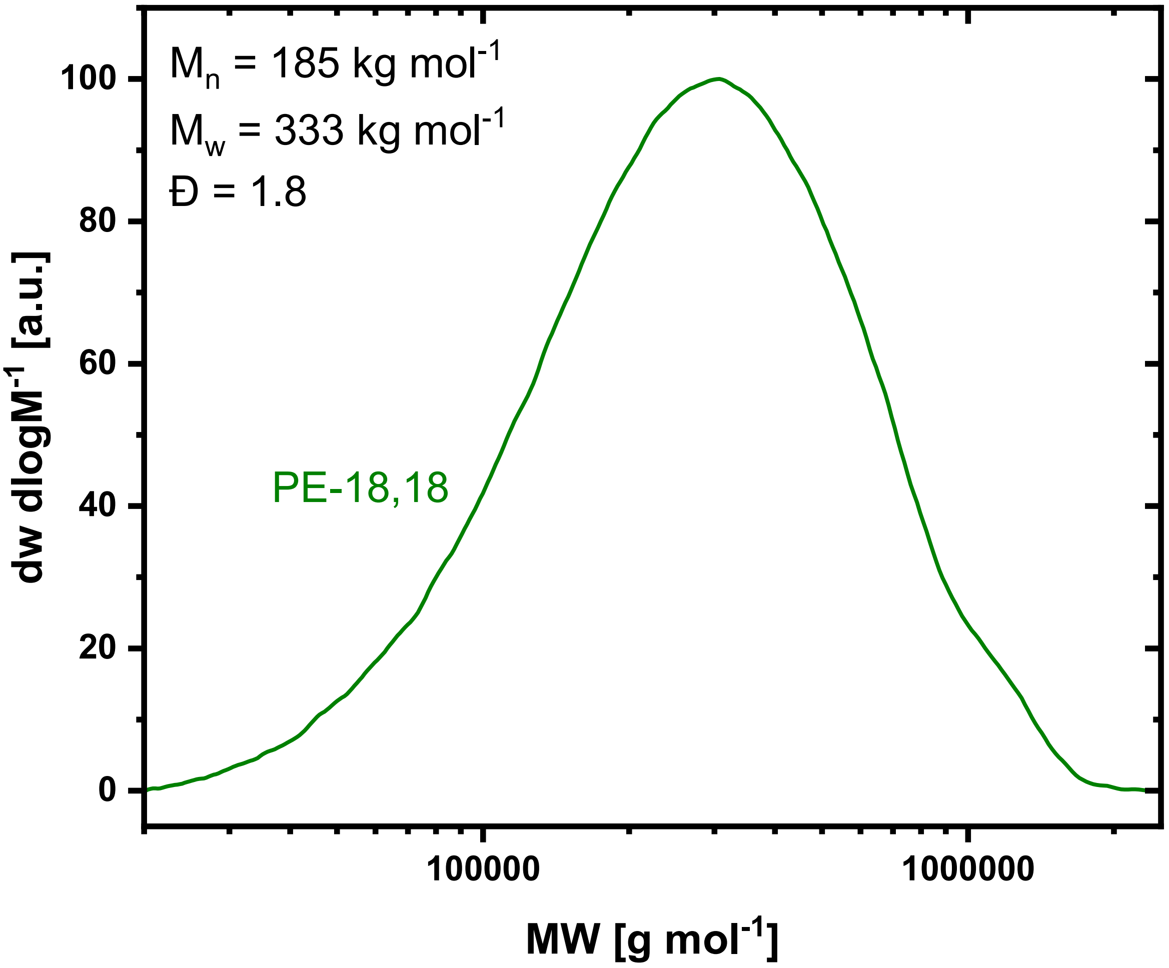


**Figure S7.** SEC trace (35 °C, chloroform) of PE-18,18 employed for melt-spinning of fibers with a *Xplore CFL 35* micro cast film line.

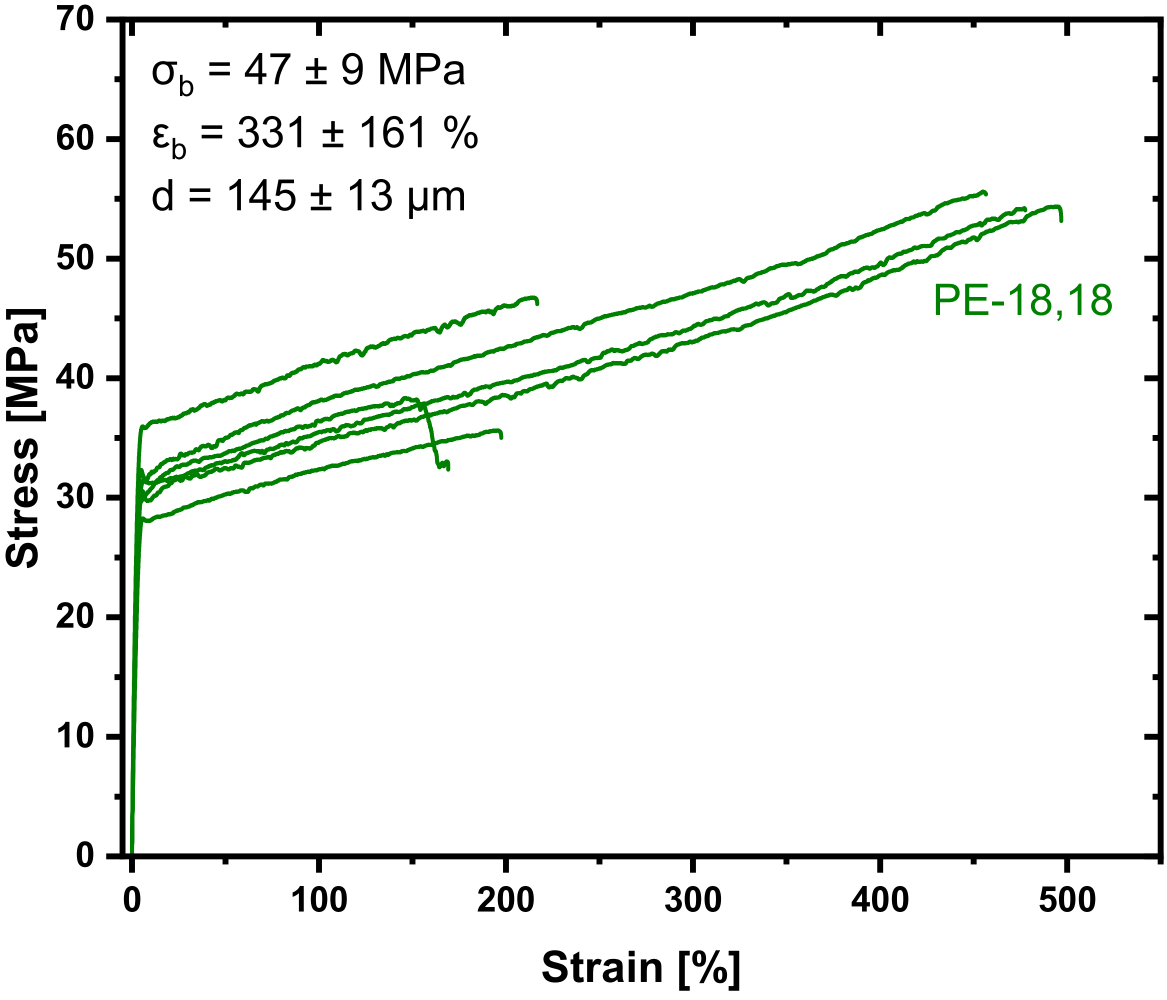


**Figure S8.** Stress strain curves of PE-18,18 fibers produced using a *Xplore MC 5* micro compounder and a *Xplore CFL 35* micro cast film line.

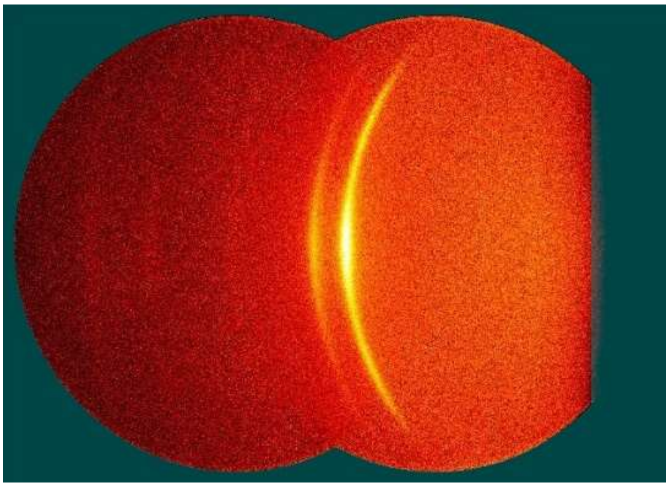

**Figure S9**. Diffraction pattern of a stretched PE-18,18 fiber from 2D detected WAXS.

**Supplementary data for PE-2,18 fibers**

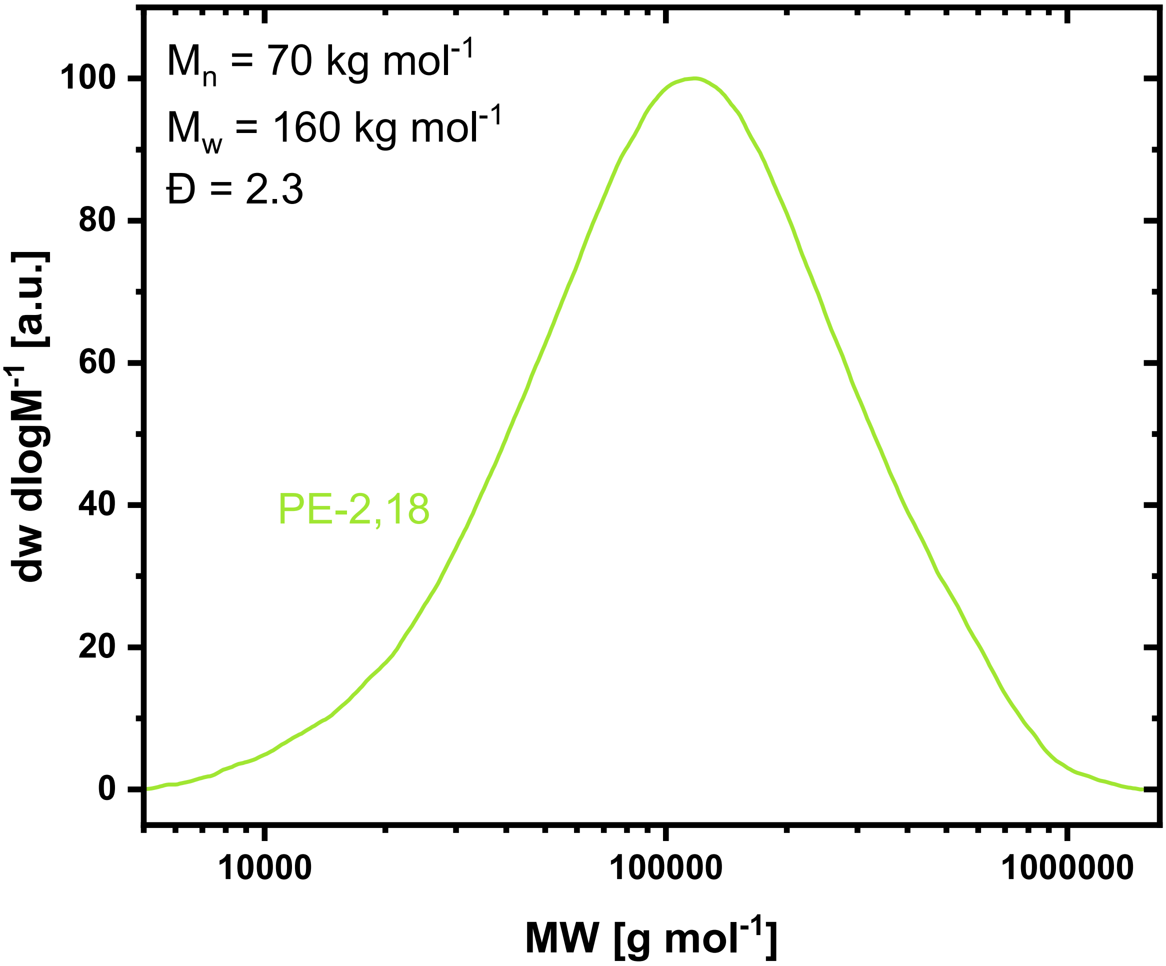


**Figure S10.** SEC trace of PE-2,18 employed in melt-spinning to monofilament.

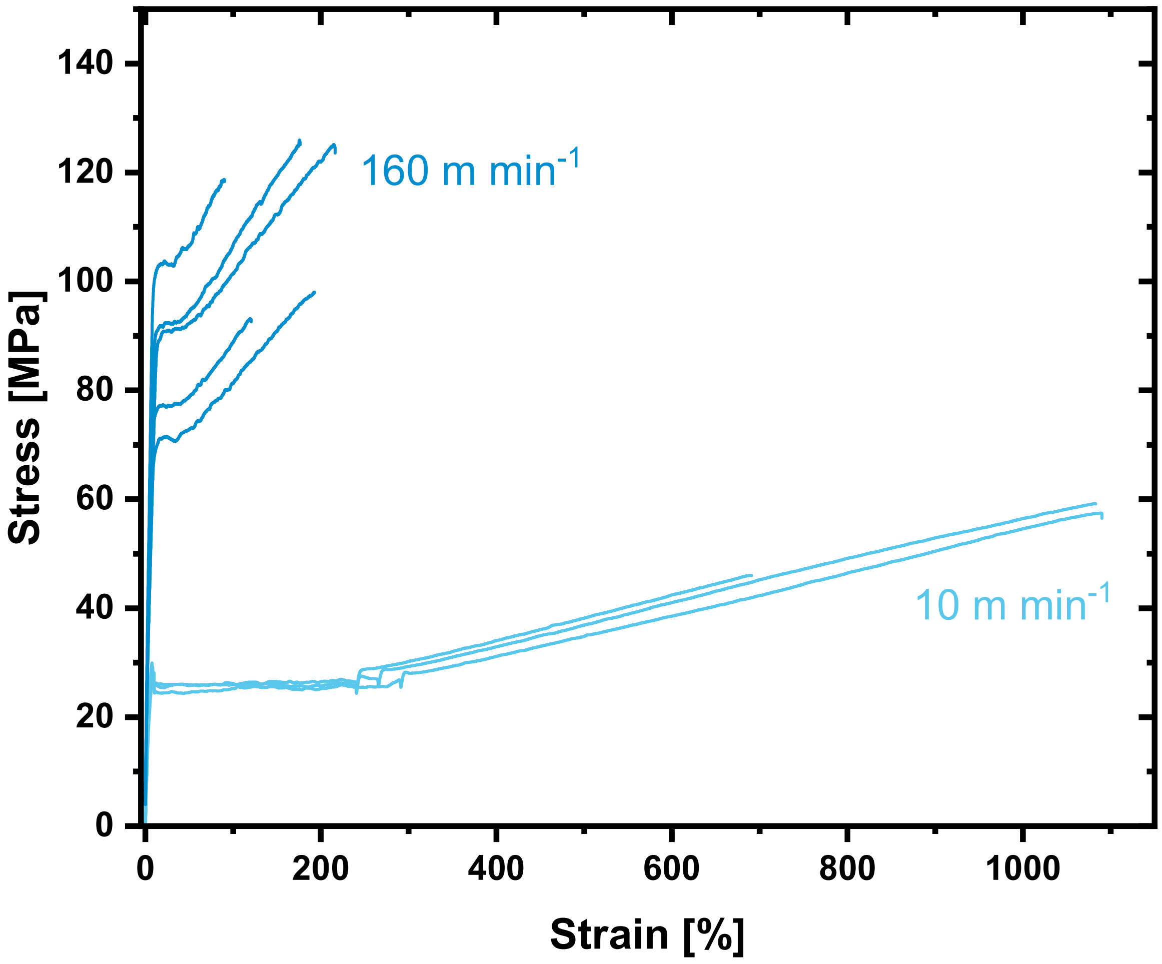


**Figure S11.** Stress-strain curves of PE-2,18 fibers spun at 10 and 160 m min$^{-1}$, respectively.

**Table S1.** Diameter (d), E-modulus (E), tensile strength ($\sigma_b$), and elongation at break ($\varepsilon_b$) of PE-2,18 fibers spun at 10 and 160 m min$^{-1}$.

| DR | d [µm] | E [MPa] | $\sigma_b$ [MPa] | $\varepsilon_b$ [%] |
|---|---|---|---|---|
| 10 m min$^{-1}$ | 239 ± 18 | 546 ± 74 | 54 ± 7 | 953 ± 228 |
| 160 m min$^{-1}$ | 50 ± 5 | 1118 ± 75 | 112 ± 16 | 158 ± 52 |

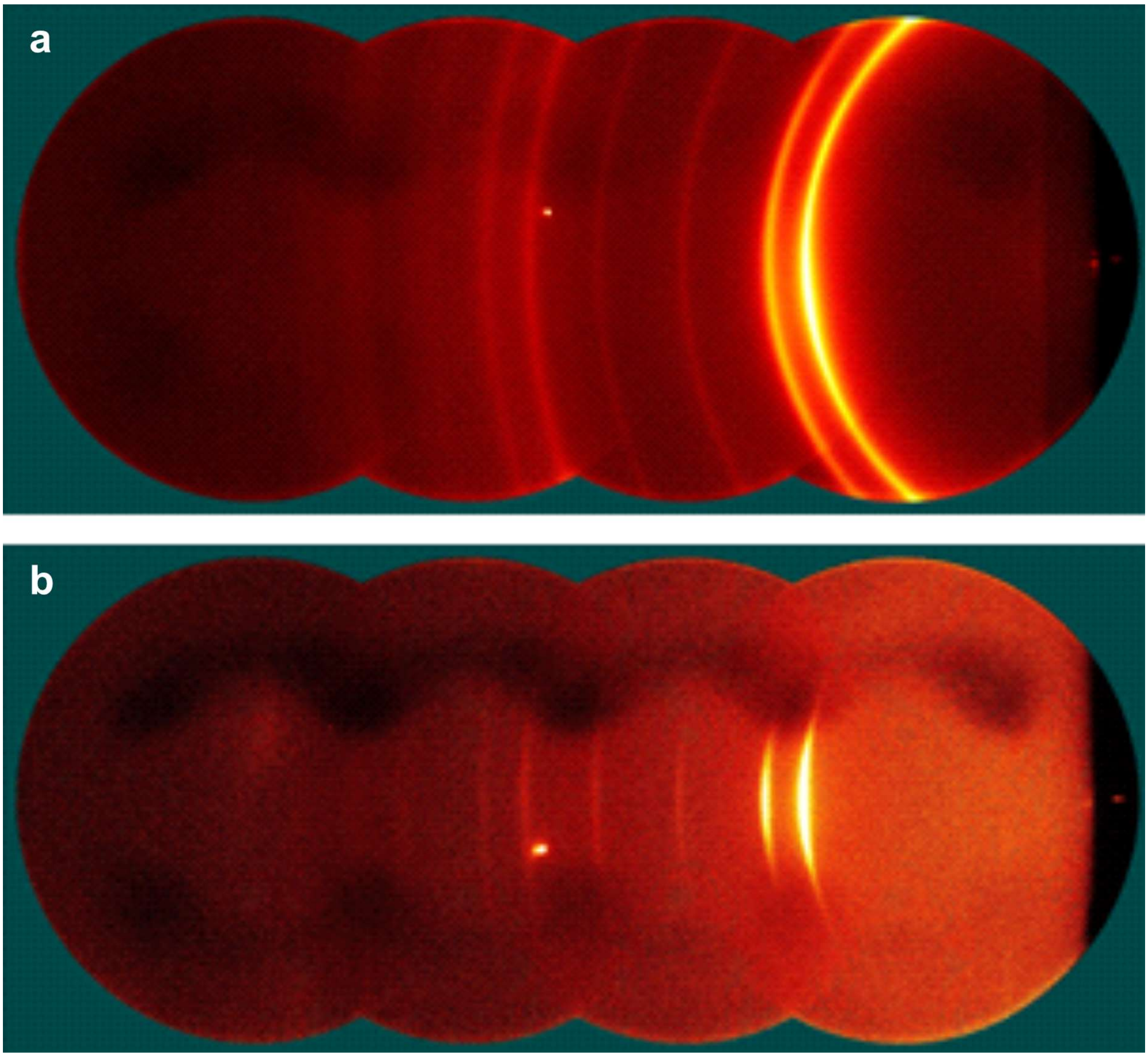


**Figure S12.** Diffraction patterns of (a) PE-2,18 granulate and (b) PE-2,18 fiber from 2D detected WAXS.

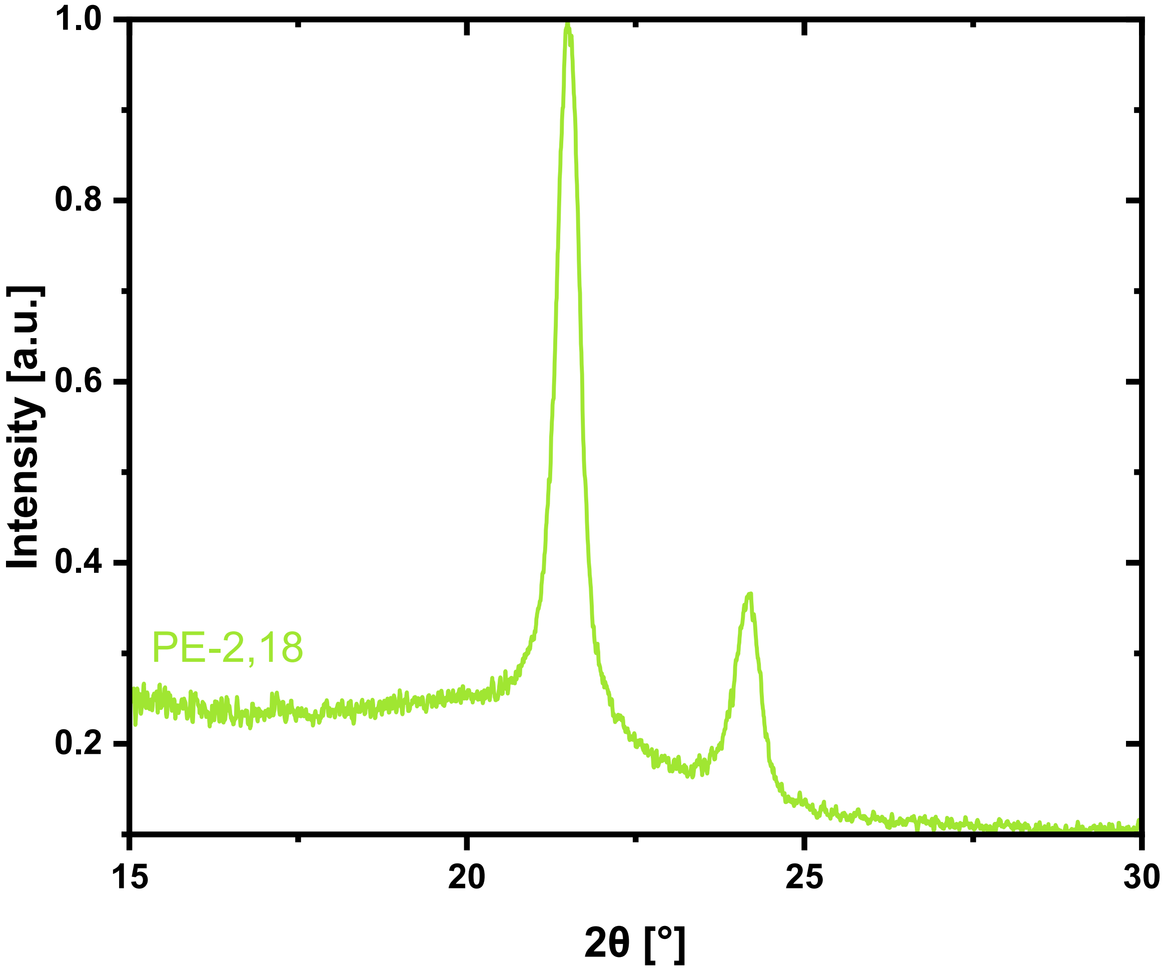


**Figure S13.** WAXS diffraction pattern of a PE-2,18 fiber.

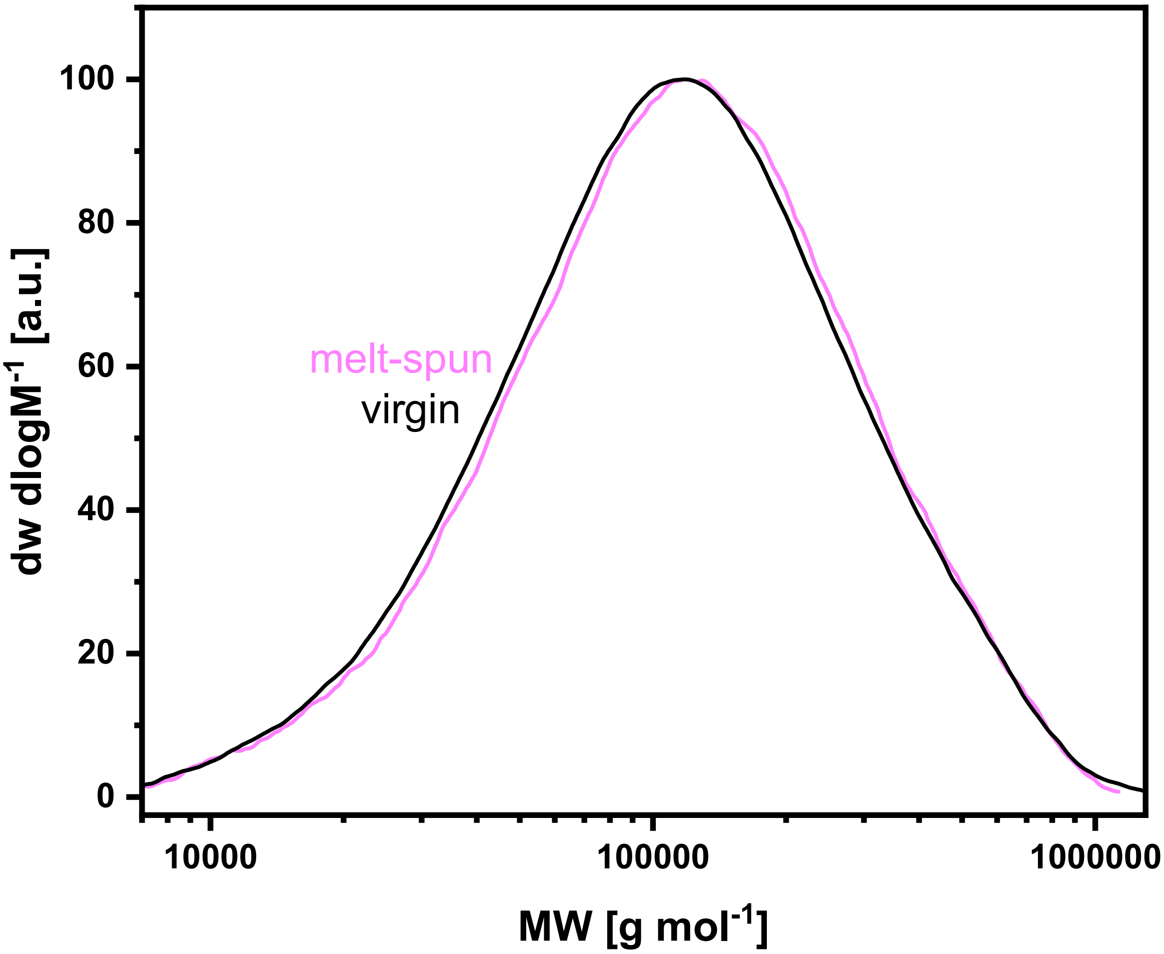


**Figure S14.** SEC traces of PE-2,18 melt-spun fibers (75 m $min^{-1}$, pink) and virgin PE-2,18 (black).

**Table S2.** Number average molecular weight ($M_n$), weight average molecular weight ($M_w$) and corresponding polydispersity index (Đ) for melt-spun PE-2,18 monofilaments from two independent runs, and virgin PE-2,18 reference as determined by SEC.

| | $M_n$ [kg $mol^{-1}$] | $M_w$ [kg $mol^{-1}$] | Đ |
|---|---|---|---|
| spun fiber expn. #1 | 73 | 163 | 2.2 |
| spun fiber expn. #2 | 80 | 179 | 2.2 |
| virgin polymer | 70 | 160 | 2.3 |

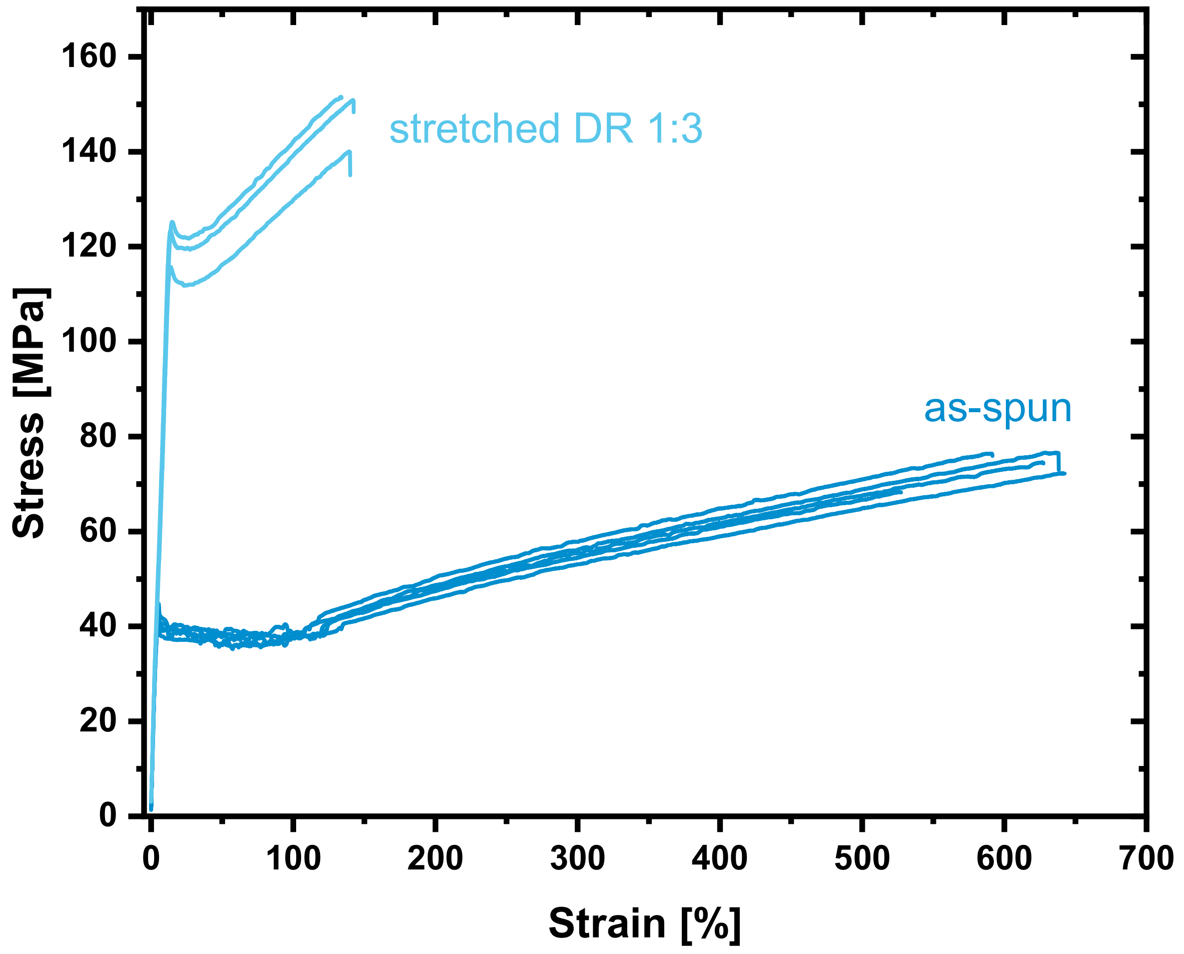


**Figure S15.** Stress-strain curves of as-spun and stretched PE-2,18 fibers.

**Table S3.** Diameter (d), E-modulus (E), tensile strength ($\sigma_b$), and elongation at break ($\varepsilon_b$) of as-spun and stretched PE-2,18 fibers.

| DR | d [µm] | E [MPa] | $\sigma_b$ [MPa] | $\varepsilon_b$ [%] |
|---|---|---|---|---|
| as-spun | 99 ± 3 | 1150 ± 40 | 74 ± 4 | 603 ± 47 |
| 1:3 | 63 ± 2 | 1017 ± 12 | 148 ± 7 | 138 ± 4 |

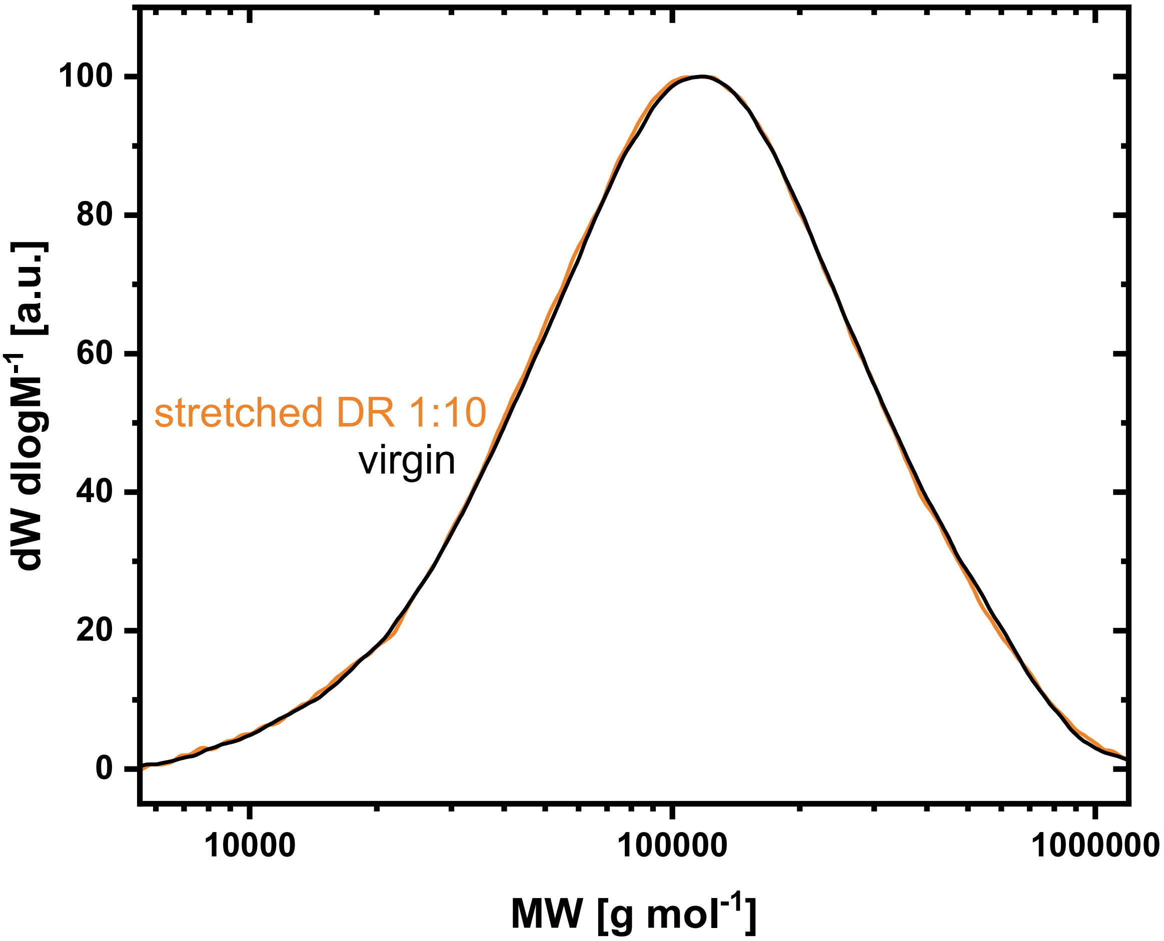


**Figure S16.** SEC traces of stretched PE-2,18 fibers (draw ratio 1:10), and virgin PE-2,18 reference.

**Table S4.** Number average molecular weight ($M_n$), weight average molecular weight ($M_w$) and corresponding polydispersity index (Đ) for PE-2,18 fibers stretched with a draw ratio 1:10, and virgin PE-2,18 as determined by SEC.

| | $M_n$ [kg mol$^{-1}$] | $M_w$ [kg mol$^{-1}$] | Đ |
|---|---|---|---|
| stretched fiber | 70 | 160 | 2.3 |
| virgin polymer | 70 | 160 | 2.3 |

**Supplementary data for enzymatic hydrolysis experiment**

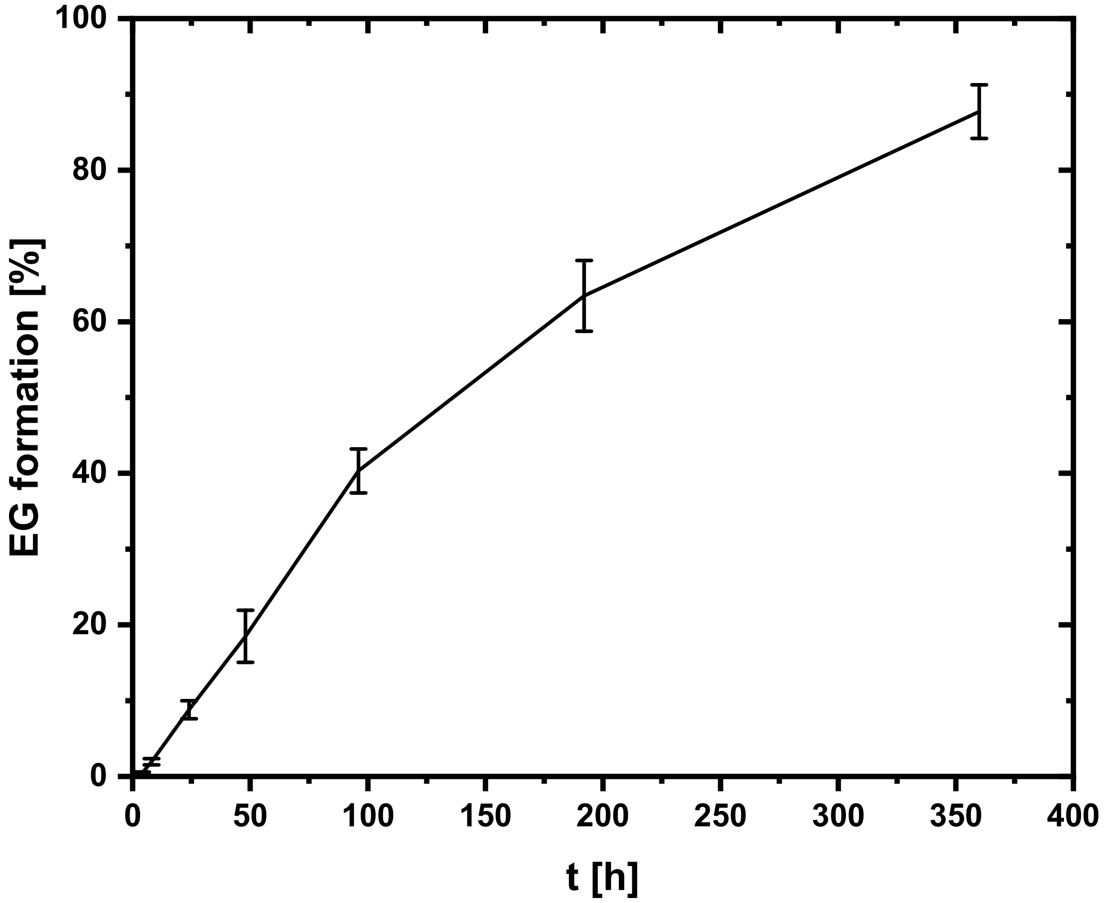


**Figure S17.** Quantification of ethylene glycol (EG) formation during hydrolysis of PE-2,18 fibers by HiC.

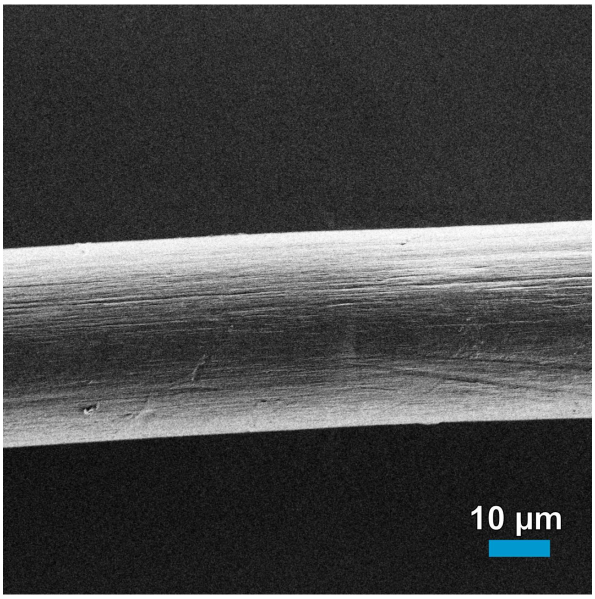


**Figure S18.** SEM image of a HDPE fiber.

## S2. Additional characterization data for PE-2,18 multifilament

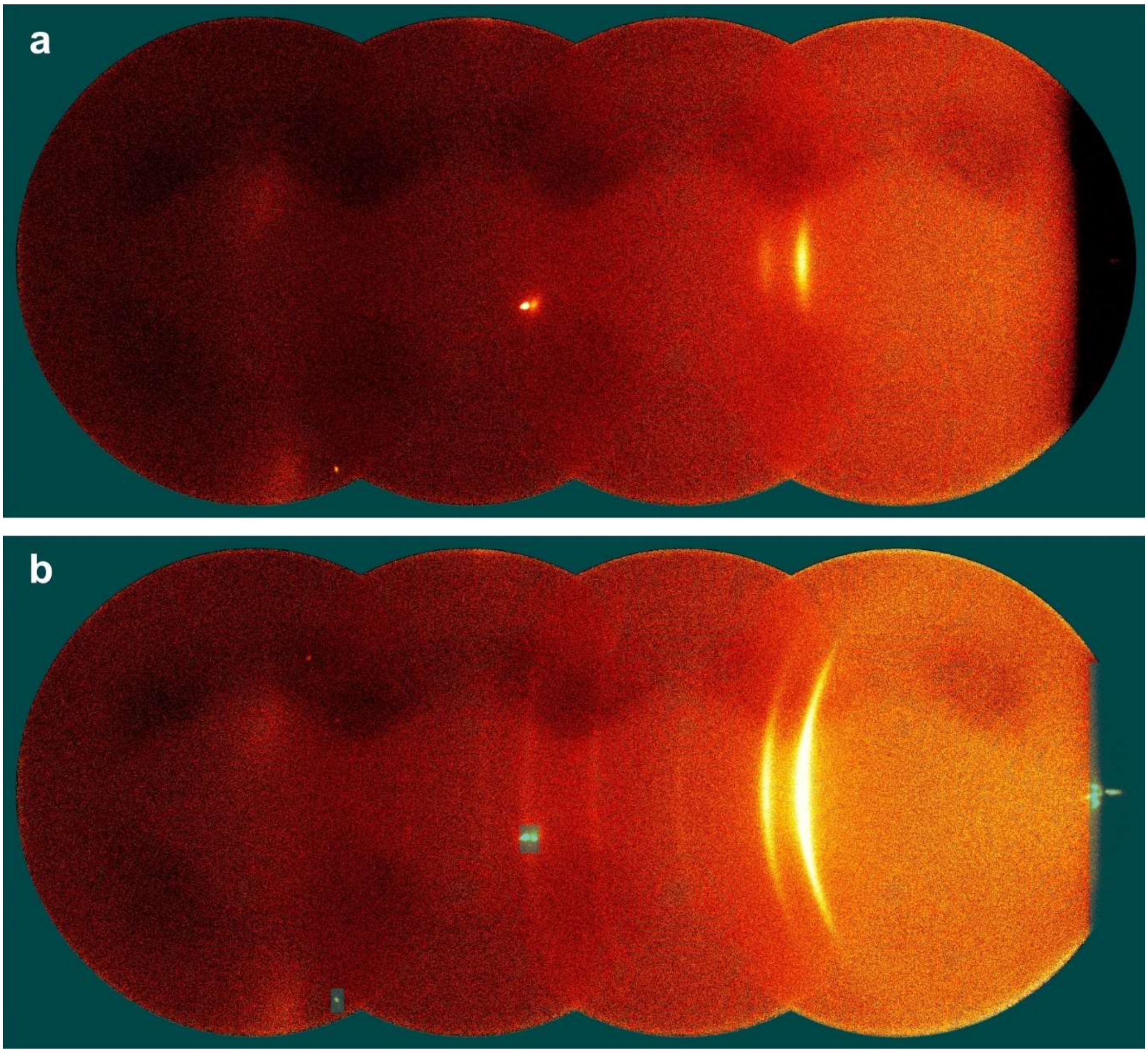


**Figure S19.** Diffraction patterns of (a) stretched PE-2,18 multifilament and (b) as-spun PE-2,18 from 2D detected WAXS.

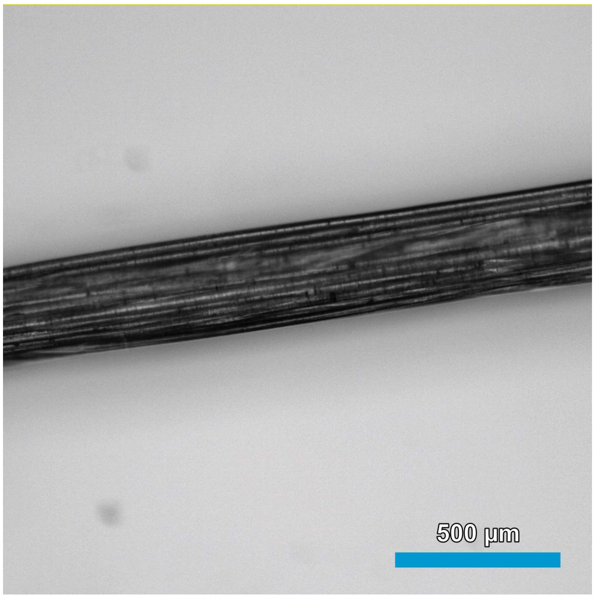


**Figure S20:** Light microscopy image of as-spun PE-2,18 multifilament.

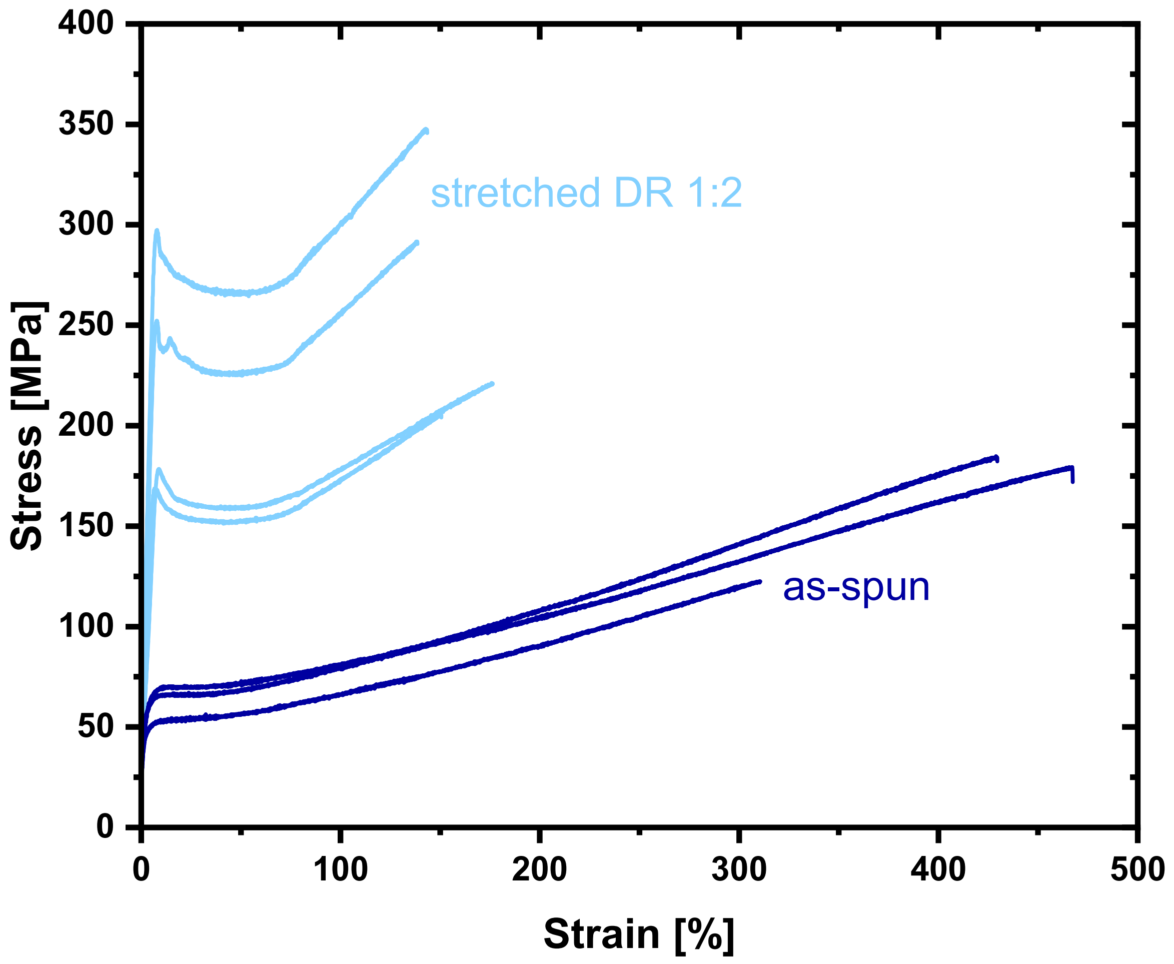


**Figure S21.** Stress-strain curves of as-spun and stretched PE-2,18 multifilament fibers.

**Table S5.** Diameter (d), E-modulus (E), tensile strength ($\sigma_b$), and elongation at break ($\varepsilon_b$) of as-spun and stretched PE-2,18 multifilament fibers.

| DR | d [µm] | E [MPa] | $\sigma_b$ [MPa] | $\varepsilon_b$ [%] |
|---|---|---|---|---|
| as-spun | 21 ± 1 | 858 ± 189 | 162 ± 35 | 402 ± 82 |
| 1:2 | 18 ± 2 | 1962 ± 566 | 267 ± 66 | 152 ± 17 |

## S3. Additional characterization data for PE-2,18 fabric

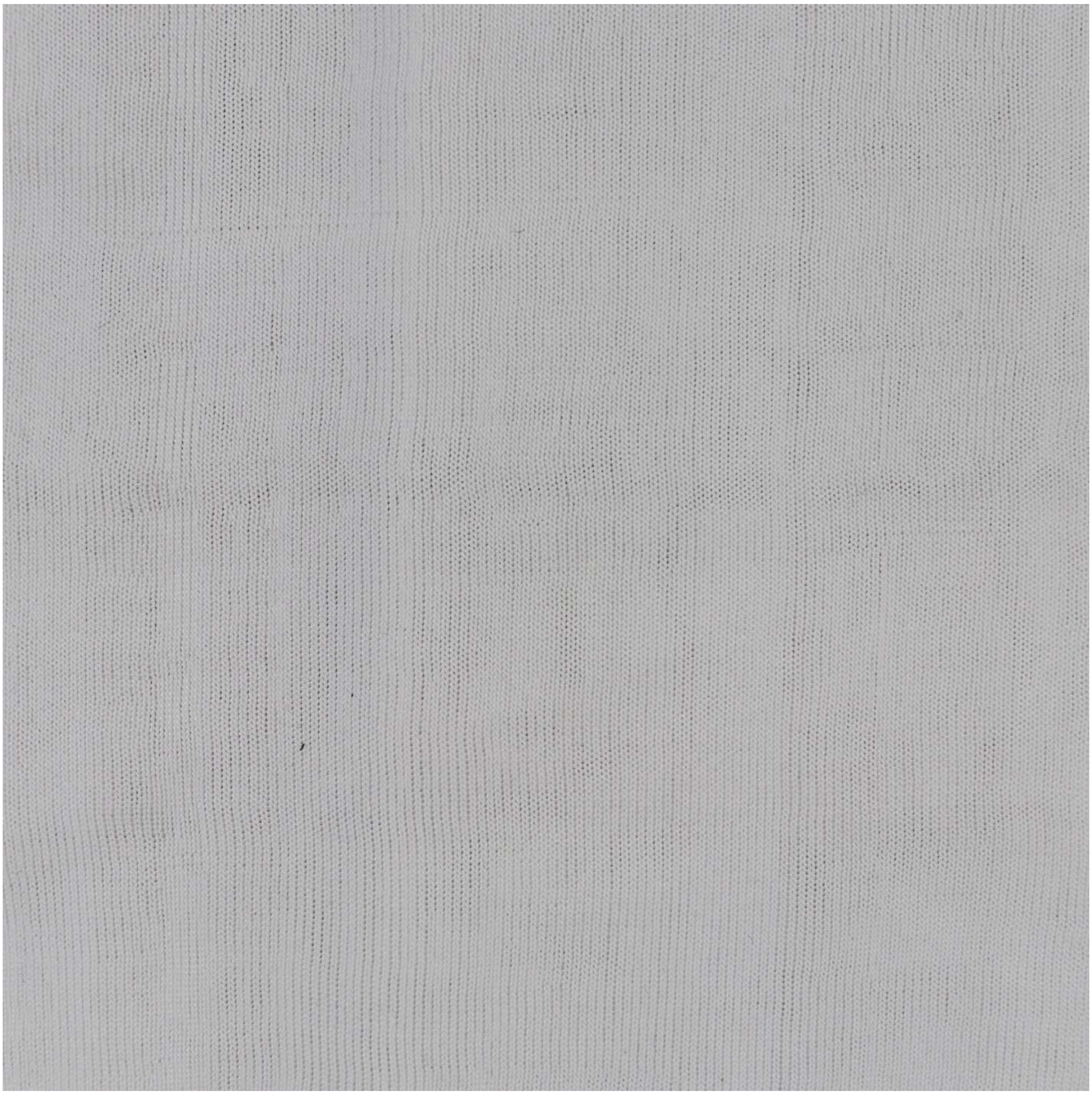

**Figure S22.** Photographic image of knit structure of PE-2,18 fabric.

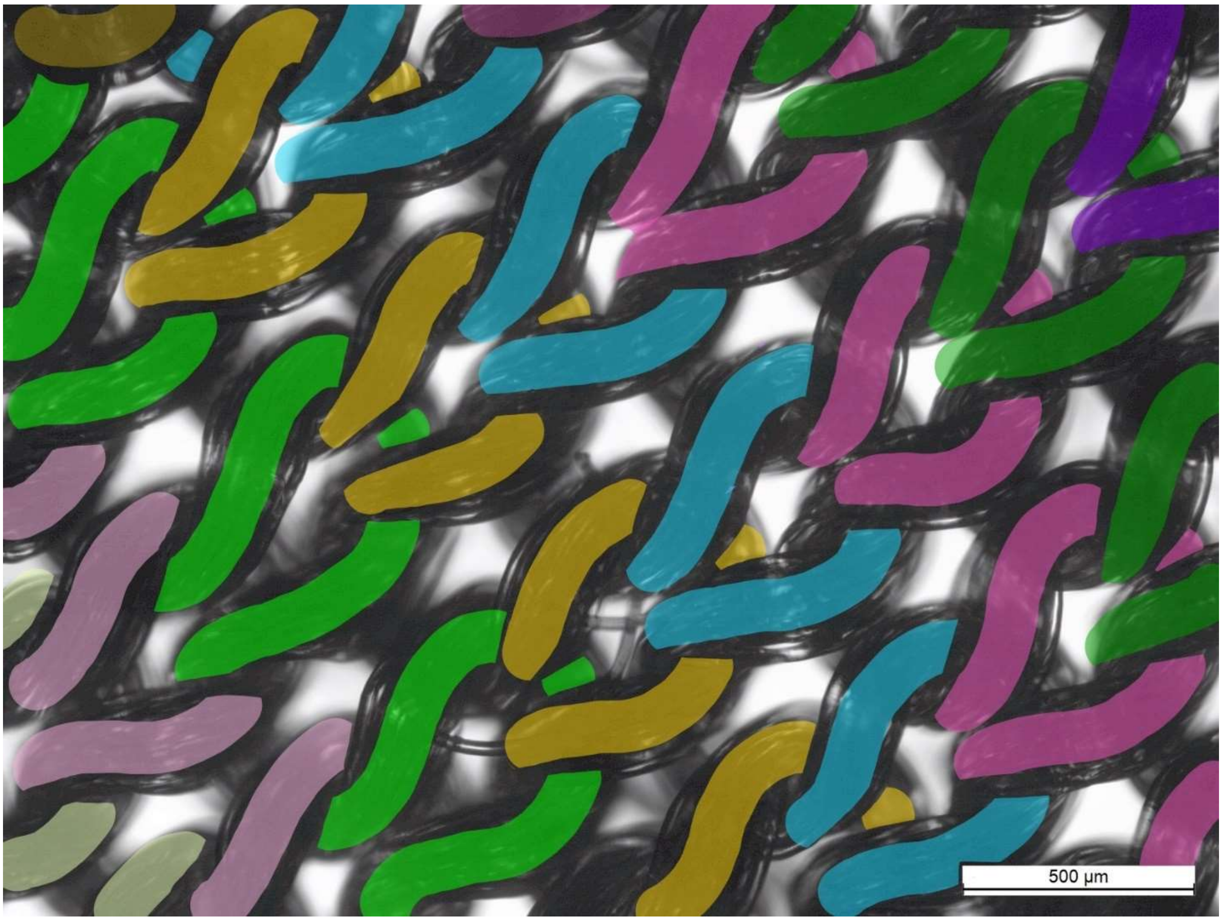


**Figure S23.** Light microscope image of knitted fabric from PE-2,18 multifilament. Loops were colored with an image editing program to better visualize the knitting pattern.

## S4. Supplementary data for machine washing experiments

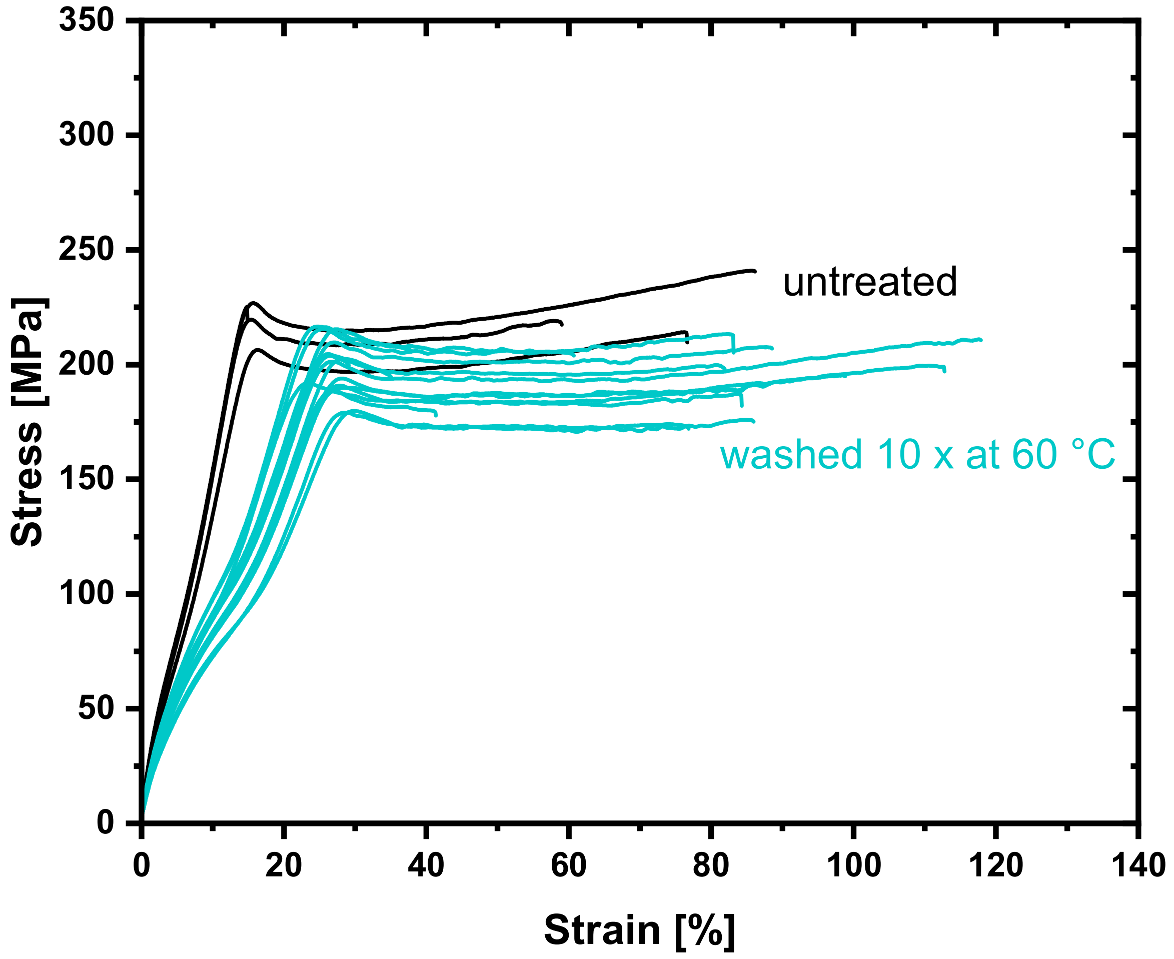


**Figure S24.** Stress-strain curves of PE-2,18 fibers washed for ten cycles at 60 °C, and reference untreated PE-2,18 fibers.

**Table S6.** Diameter (d), tensile strength ($\sigma_b$), elongation at break ($\varepsilon_b$) and E-modulus of PE-2,18 fibers washed at 60 °C and untreated PE-2,18 reference fibers as determined by tensile testing.

| | d [µm] | $\sigma_b$ [MPa] | $\varepsilon_b$ [%] | E [MPa] |
|---|---|---|---|---|
| fiber washed 10 x at 60 °C | 58 ± 2 | 200 ± 12 | 47 ± 35 | 1342 ± 150 |
| untreated fiber | 50 ± 1 | 225 ± 12 | 48 ± 38 | 1954 ± 128 |

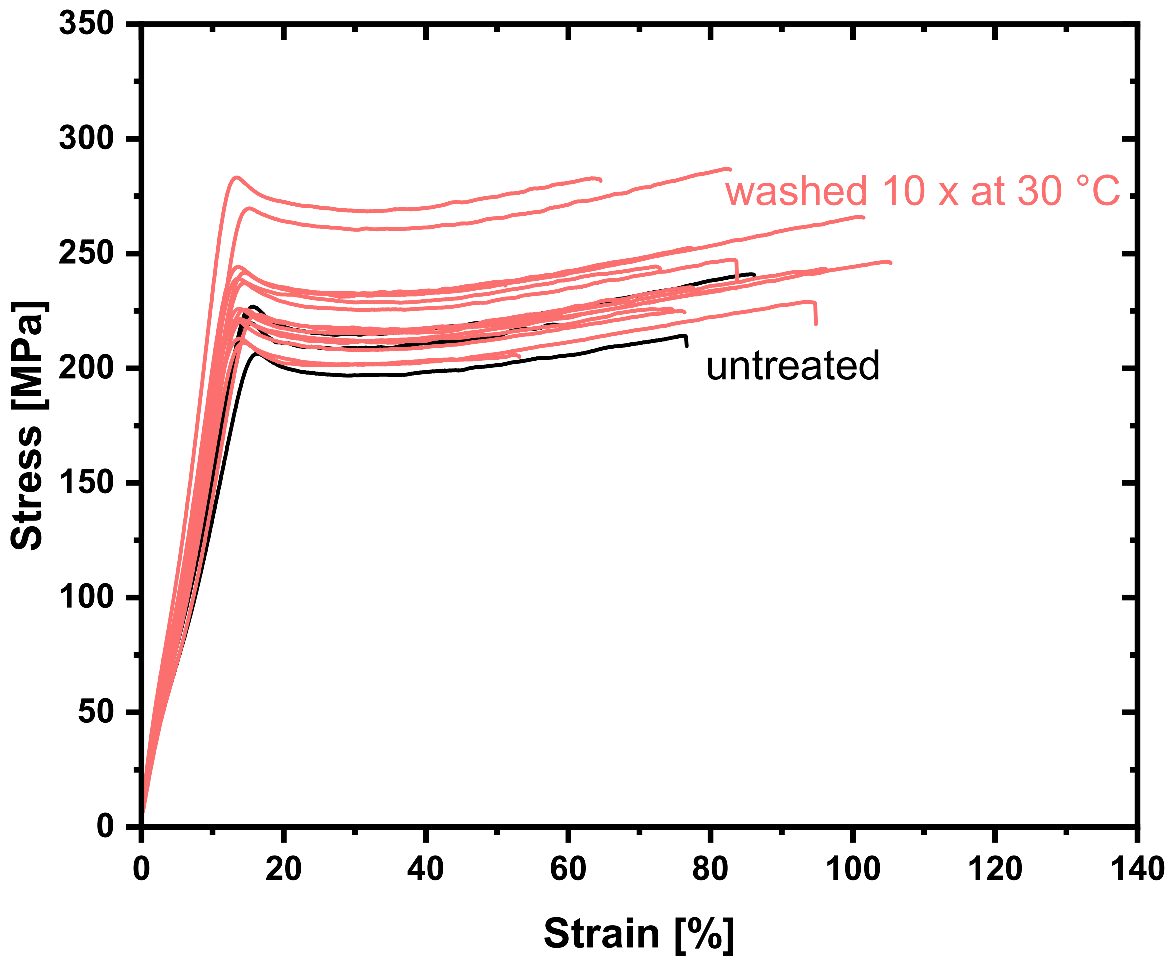


**Figure S25.** Stress-strain curves of PE-2,18 fibers washed for ten cycles at 30 °C, and untreated PE-2,18 fibers as reference.

**Table S7.** Diameter (d), tensile strength ($\sigma_b$), elongation at break ($\varepsilon_b$) and E-modulus of PE-2,18 fibers washed at 30 °C and untreated PE-2,18 reference fibers as determined by tensile testing.

| | d [µm] | $\sigma_b$ [MPa] | $\varepsilon_b$ [%] | E [MPa] |
|---|---|---|---|---|
| fiber washed 10 x at 30 °C | 50 ± 2 | 245 ± 20 | 72 ± 30 | 1900 ± 265 |
| untreated fiber | 50 ± 1 | 225 ± 12 | 48 ± 38 | 1954 ± 128 |

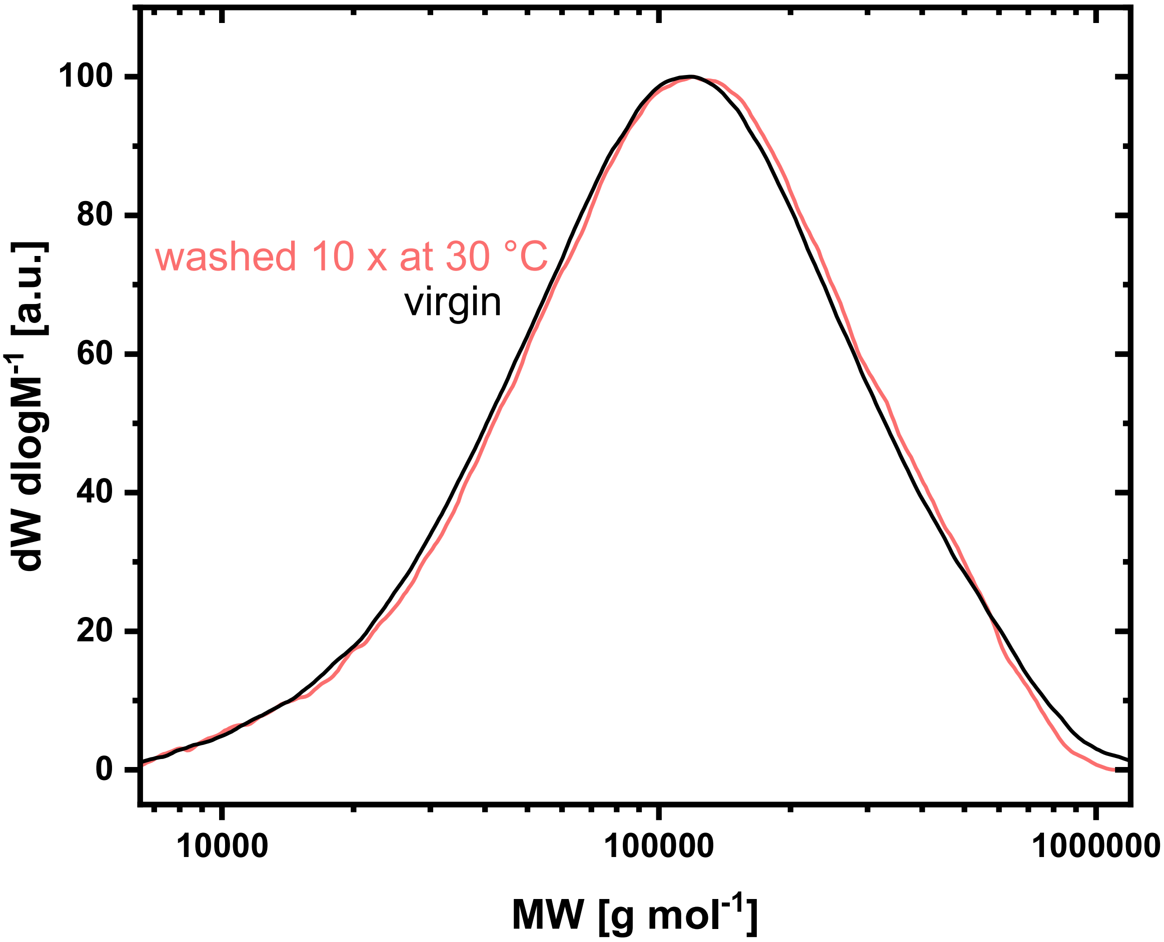


**Figure S26.** SEC traces of PE-2,18 fibers after ten cycles of washing at 30 °C, and virgin PE-2,18.

**Table S8.** Number average molecular weight ($M_n$), weight average molecular weight ($M_w$) and resulting polydispersity index (Đ) for PE-2,18 fibers washed at 30 °C and virgin PE-2,18 reference as determined by SEC.

| | $M_n$ [kg mol$^{-1}$] | $M_w$ [kg mol$^{-1}$] | Đ |
|---|---|---|---|
| fiber washed 10 x at 30 °C | 72 | 159 | 2.2 |
| virgin polymer | 70 | 160 | 2.3 |

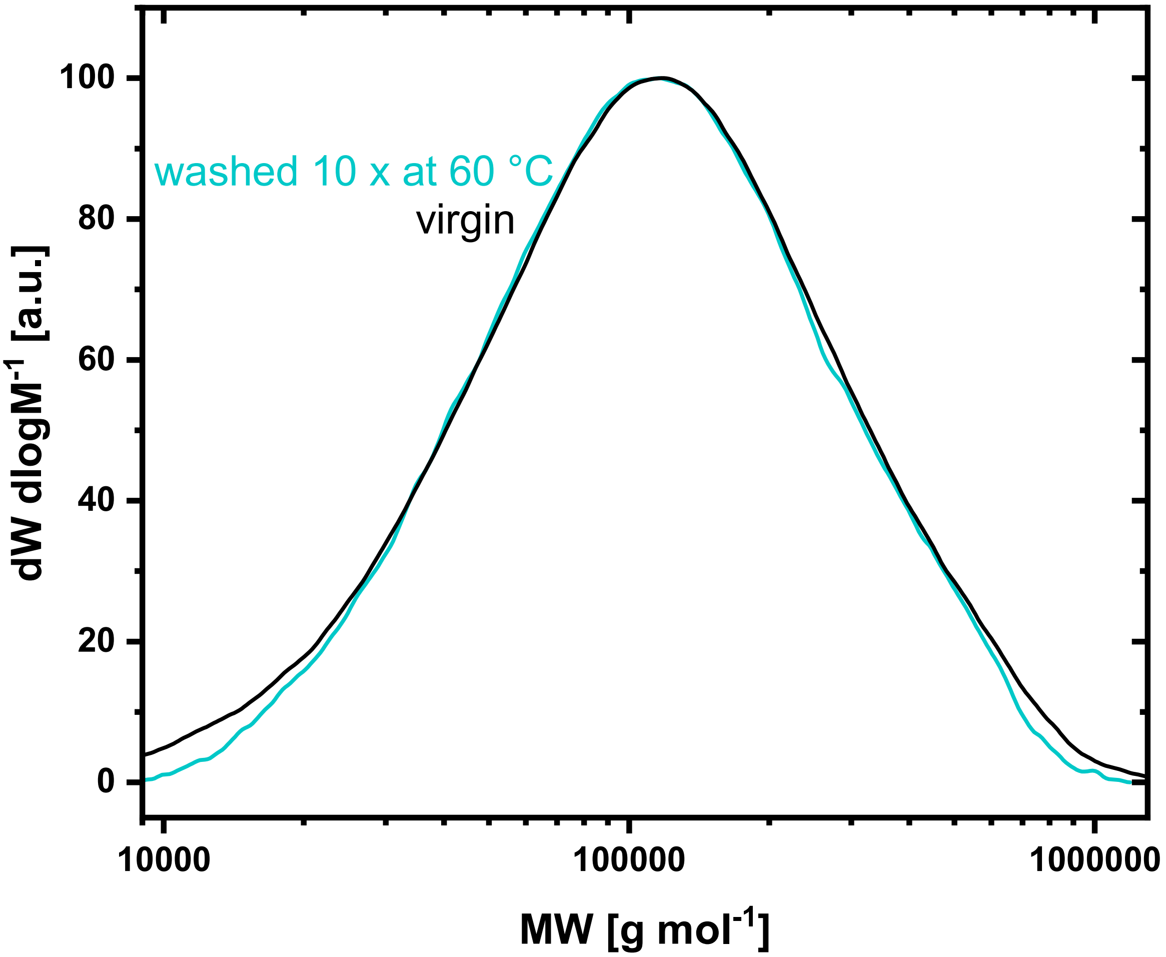


**Figure S27.** SEC traces of PE-2,18 fibers after ten cycles of washing at 60 °C and untreated virgin PE-2,18.

**Table S9.** Number average molecular weight ($M_n$), weight average molecular weight ($M_w$) and resulting polydispersity index (Đ) for PE-2,18 fibers washed at 60 °C and virgin PE-2,18 reference as determined by SEC.

| | $M_n$ [kg mol$^{-1}$] | $M_w$ [kg mol$^{-1}$] | Đ |
|---|---|---|---|
| fiber washed 10 x at 60 °C | 77 | 156 | 2.0 |
| virgin polymer | 70 | 160 | 2.3 |

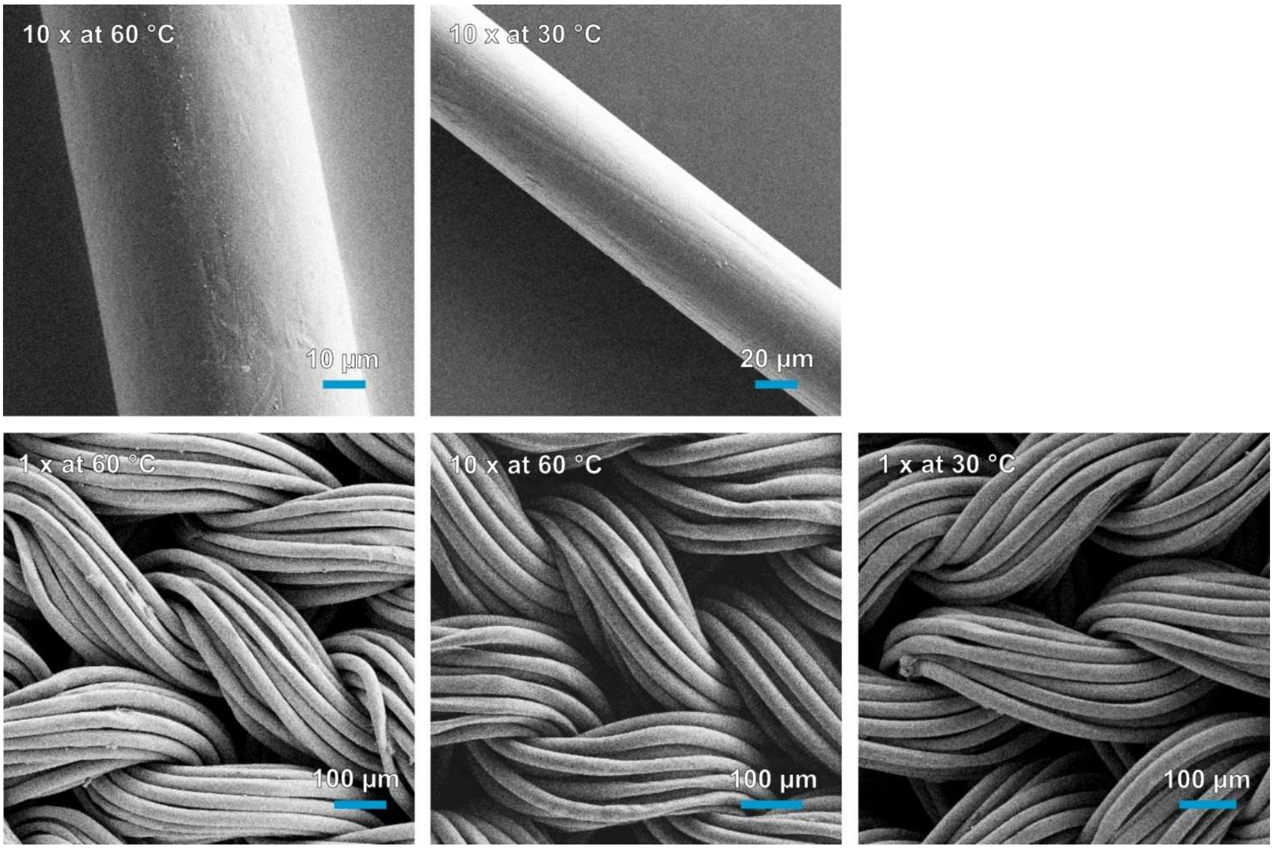


**Figure S28.** SEM images of PE-2,18 fibers and fabric washed for ten cycles at 30 and 60 °C, respectively.

## S5. Additional characterization data for PA-6,18

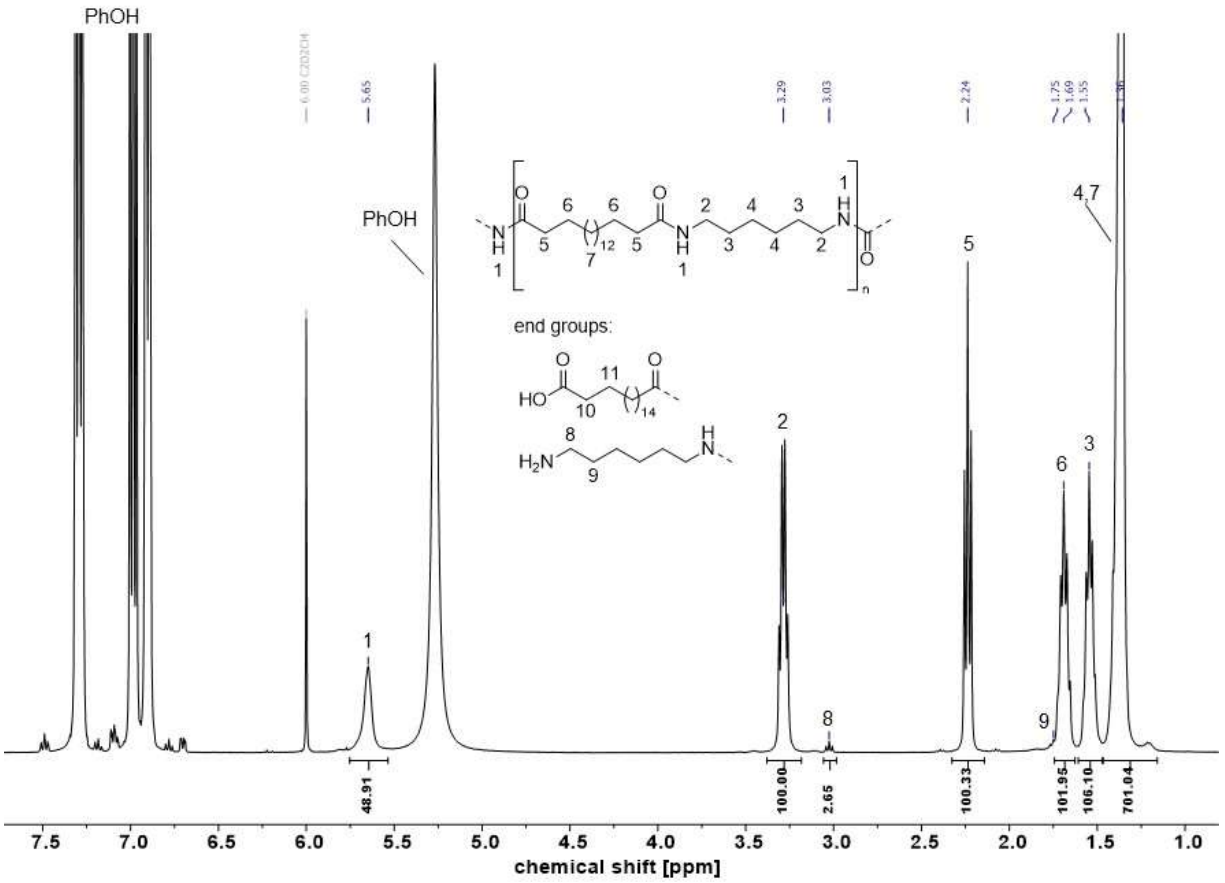


**Figure S29.** $^1$H NMR spectrum of PA-6,18. Note that no acid end group (10 and 11) is apparent.

$^1$H NMR (400 MHz, $C_2D_2Cl_4$/PhOH, 373 K, ppm): $\delta$ = 5.65 (s, 50H, H-1), 3.29 (q, $^3J_{H,H}$ = 6.5 Hz, 100H, H-2), 3.03 (t, $^3J_{H,H}$ = 7.1 Hz, 2.65H, *end group* H-8), 2.24 (t, $^3J_{H,H}$ = 7.6 Hz, 100H, H-5), 1.75 (m, *n.a.*, H-9), 1.69 (quint, $^3J_{H,H}$ = 7.2 Hz, 100H, H-6), 1.55 (quint, $^3J_{H,H}$ = 6.8 Hz, 100H, H-3), 1.36 (m, 700H, H-4 and H-7).

$$M_n = \frac{\int 2}{\int 8 + \int 10} \times M_{rep.unit}$$

**Equation S1.** Determination of $M_n$ for aliphatic polyamides via end group analysis in $^1$H NMR spectra. For PA-6,18 $M_{rep.unit}$ = 394. 64 g mol$^{-1}$.

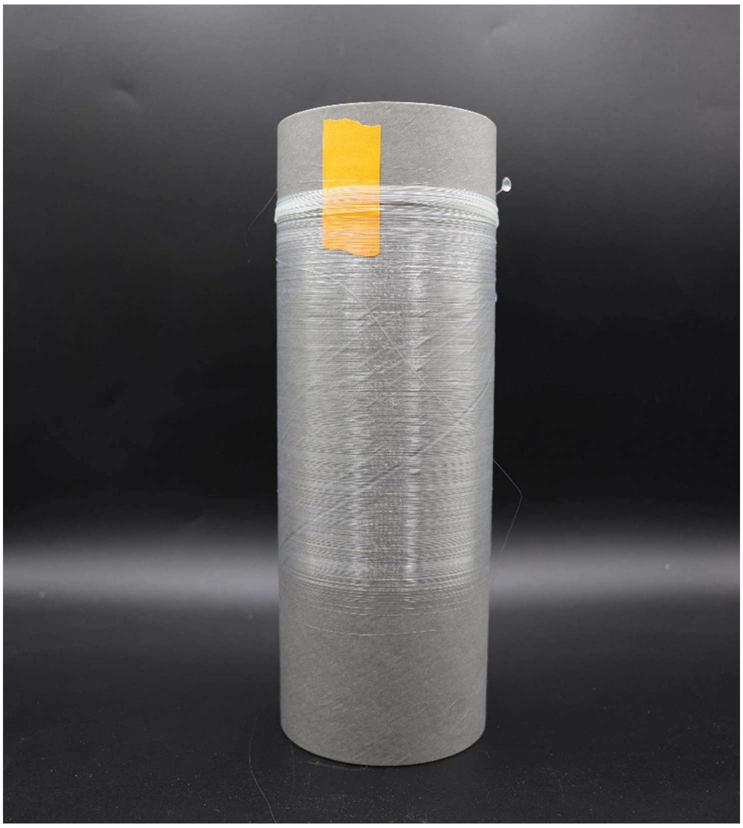

**Figure S30.** PA-6,18 fiber on carton bobbin. A continuous fiber of 150 m length was spun with a winding torque of 50 Nmm.

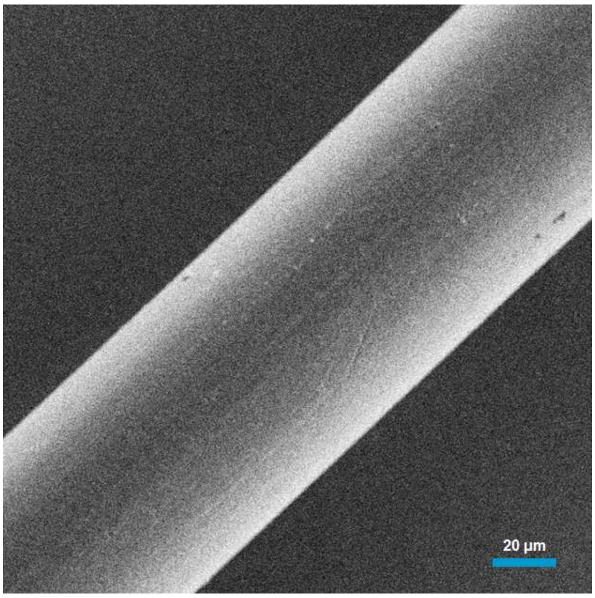


**Figure S31.** SEM image of a PA-6,18 fiber.

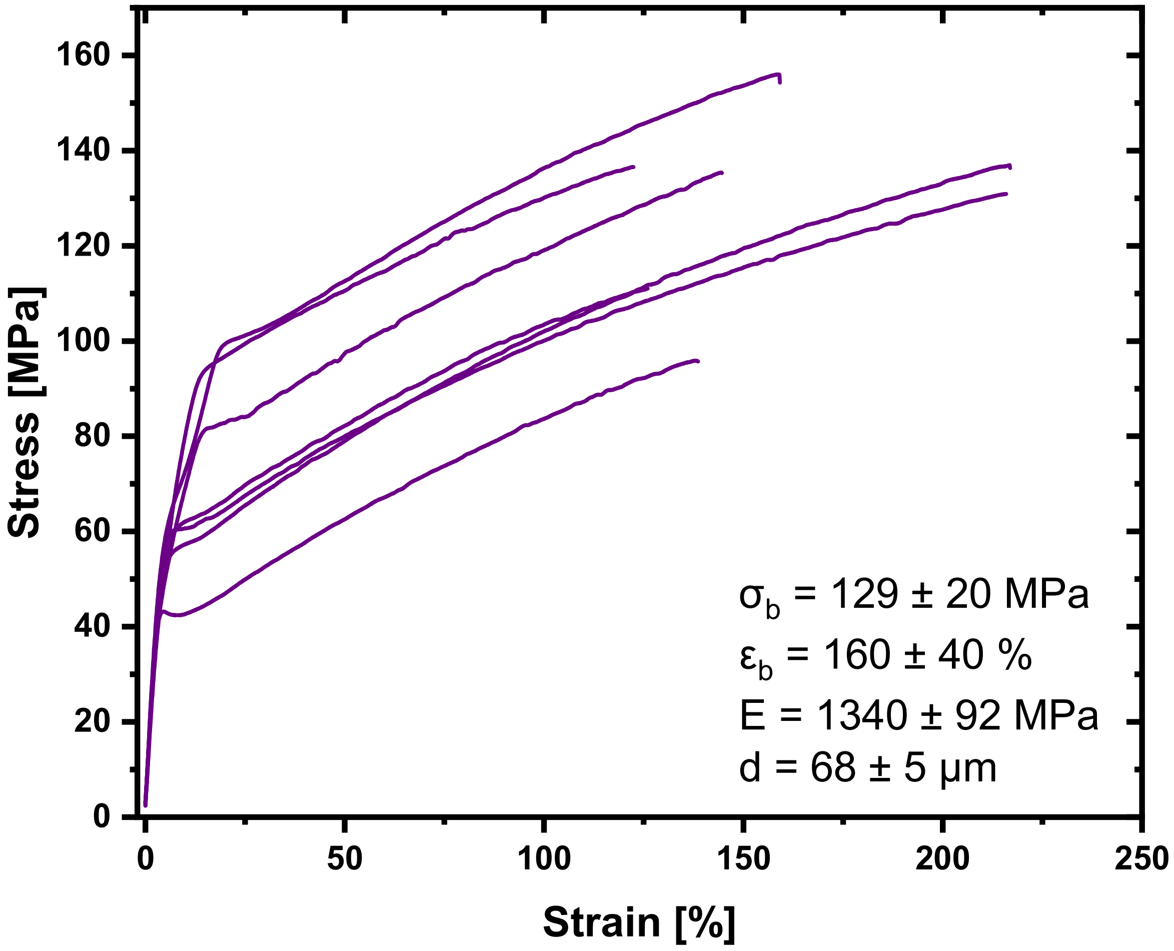


**Figure S32.** Stress-strain curves of PA-6,18 fibers.
